# Low Levels of p53 Protein and Chromatin Silencing of p53 Target Genes Repress Apoptosis in *Drosophila* Endocycling Cells

**DOI:** 10.1371/journal.pgen.1004581

**Published:** 2014-09-11

**Authors:** Bingqing Zhang, Sonam Mehrotra, Wei Lun Ng, Brian R. Calvi

**Affiliations:** Department of Biology, Indiana University, Bloomington, Indiana, United States of America; The University of North Carolina at Chapel Hill, United States of America

## Abstract

Apoptotic cell death is an important response to genotoxic stress that prevents oncogenesis. It is known that tissues can differ in their apoptotic response, but molecular mechanisms are little understood. Here, we show that *Drosophila* polyploid endocycling cells (G/S cycle) repress the apoptotic response to DNA damage through at least two mechanisms. First, the expression of all the *Drosophila* p53 protein isoforms is strongly repressed at a post-transcriptional step. Second, p53-regulated pro-apoptotic genes are epigenetically silenced in endocycling cells, preventing activation of a paused RNA Pol II by p53-dependent or p53-independent pathways. Over-expression of the p53A isoform did not activate this paused RNA Pol II complex in endocycling cells, but over-expression of the p53B isoform with a longer transactivation domain did, suggesting that dampened p53B protein levels are crucial for apoptotic repression. We also find that the p53A protein isoform is ubiquitinated and degraded by the proteasome in endocycling cells. In mitotic cycling cells, p53A was the only isoform expressed to detectable levels, and its mRNA and protein levels increased after irradiation, but there was no evidence for an increase in protein stability. However, our data suggest that p53A protein stability is regulated in unirradiated cells, which likely ensures that apoptosis does not occur in the absence of stress. Without irradiation, both p53A protein and a paused RNA pol II were pre-bound to the promoters of pro-apoptotic genes, preparing mitotic cycling cells for a rapid apoptotic response to genotoxic stress. Together, our results define molecular mechanisms by which different cells in development modulate their apoptotic response, with broader significance for the survival of normal and cancer polyploid cells in mammals.

## Author Summary

In order to maintain genome integrity, eukaryotic cells have evolved multiple ways to respond to DNA damage stress. One of the major cellular responses is apoptosis, during which the cell undergoes programmed cell death in order to prevent the propagation of the damaged genome to daughter cells. Although clinical observations and other studies have shown that tissues can differ in their apoptotic response, the molecular mechanisms underlying these differences are largely unknown. We have shown in our model system, *Drosophila*, that endocycling cells do not initiate cell death in response to DNA damage. The endocycle is a cell cycle variation that is widely found in nature and conserved from plant to animals. During the endocycle, cells duplicate their genomic DNA but do not enter mitosis to segregate chromosomes, resulting in a polyploid genome content. In this study, we investigate how the apoptotic response to DNA damage is repressed in endocycling cells. We find that the *Drosophila* ortholog of the human p53 tumor suppressor protein is expressed at very low levels in endocycling cells. Moreover, the downstream pro-apoptotic genes that are regulated by p53 are epigenetically silenced in endocycling cells. Our results provide important insights into tissue-specific apoptotic responses in development, with possible broader impact on understanding radiation therapy response and cancer of different tissues.

## Introduction

Eukaryotic cells respond to DNA damage via multiple pathways. Checkpoints arrest the cell cycle and mobilize the DNA repair machinery to fix the damage [1], [2]. If this genotoxic stress is severe, however, the cells can enter a quiescent state known as senescence, or initiate programmed cell death (PCD), with one important type called apoptosis [3], [4]. Mutations in genes that compromise these pathways result in genome instability and cancer [5]. Cells from different tissues respond to DNA damage in different ways, but the mechanism(s) underlying this difference among tissues remains poorly characterized [6], [7]. Here, we use *Drosophila* as a model to define the mechanisms by which cells in development differ in their apoptotic response to genotoxic stress.

Many investigations have focused on how cells in culture respond to genotoxic stress, but much less is known about the different stress responses of tissues *in vivo* [7]–[9]. It is known that some tissues in mice and humans have higher levels of apoptosis in response to the genotoxic stress of ionizing radiation, which is largely dependent on the p53 tumor suppressor [9]–[11]. Other studies have described the transcriptional outputs downstream of p53 in different tissues in response to radiation [12]–[17]. Very little is known, however, about the molecular mechanisms that determine these tissue-specific activities of p53 and the apoptotic response to ionizing radiation [7], [18]. Defining the mechanisms of the tissue-specific apoptotic response is important to fully understand the sensitivity of different tumors to radiation therapy and the deleterious effects of this therapy on healthy tissues.

We had previously shown that *Drosophila* tissues that are composed of cells in the endocycle repress the apoptotic response to DNA damage [19]. The endocycle is a cell cycle variation with alternating gap (G) and DNA synthesis (S) phases without mitosis (M), and results in large, polyploid cells [20]–[22]. Cells switch into polyploid cell cycles as a part of normal development or regeneration in a wide variety of organisms including humans [20]. In addition, emerging evidence suggests that cancer cells polyploidize by inappropriately switching into an endocycle, which may contribute to genome instability and oncogenesis [23]–[25].

Our previous evidence suggested that *Drosophila* endocycling cells do not apoptose in response to DNA damage because both p53-dependent and independent pathways are repressed [19]. During the normal response to DNA damage, the ATM checkpoint kinase phosphorylates the Chk2 kinase, which in turn phosphorylates p53 [26]–[31]. Phosphorylation of p53 induces transcription of a number of downstream genes, including pro-apoptotic genes at one large complex locus called H99 [32]–[37]. In most tissues, the expression of the H99 genes, *reaper (rpr)*, *Head involution defective (Hid)*, and *sickle (skl)* are dependent on p53 after DNA damage, and evidence suggests that at least *rpr* is a direct target of p53 [3], [29], [38]. In endocycling cells, however, we found that the expression of the H99 genes was 10–100's of fold lower relative to mitotic cells, despite activation of the upstream ATM kinase by DNA damage [19], [39]. Endocycling cells also did not have a delayed apoptotic response to DNA damage, which in other tissues is mediated by p53-independent pathways that induce *Hid* expression [19], [40]–[43]. Together, this evidence suggested that the block to apoptosis in the endocycling cells is at or upstream of H99 gene expression, but the mechanism remained unknown.

In this study, we investigate the molecular mechanisms underlying the different apoptotic responses of mitotic cycling and endocycling cells. Our evidence suggests that p53-dependent and p53-independent apoptotic pathways are repressed in endocycling cells through epigenetic silencing of pro-apoptotic genes at the H99 locus. In addition, we find that although p53 mRNA levels are comparable between mitotic cycling and endocycling cycling cells, p53 protein is undetectable in endocycling cells. Thus, similar to humans, *Drosophila* p53 protein levels are regulated. In mitotic cycling cells, p53 protein and a paused RNA polymerase complex at the H99 gene promoters may prepare these cells to rapidly respond to genotoxic stress. We discuss the significance of these results in the context of tissue-specific apoptotic responses in development and cancer.

## Results

### H99 genes *rpr* and *hid* are transcriptionally repressed in endocycling cells

**Figure 1:**
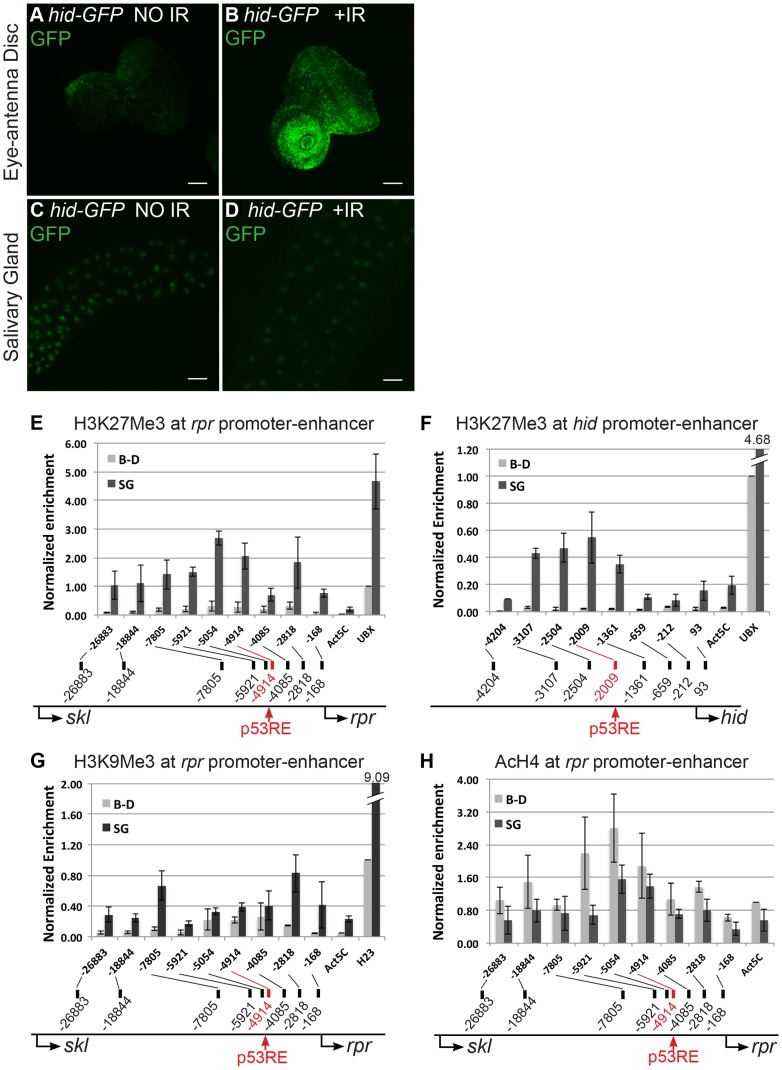
Pro-apoptotic genes at the H99 locus are transcriptionally silent and have repressive chromatin marks in endocycling cells. (A–D) Expression of the *hid-GFP* promoter-reporter in 3^rd^ instar, mitotic cycling eye-antennal discs (A,B) or endocycling salivary glands (C,D) without (A,C) or with (B,D) 4,000 rads of IR (Scale bars are 100 microns). Other mitotic cycling tissues, e.g. larval brain and wing/leg imaginal discs, showed similar GFP expression patterns. (E–H) ChIP-qPCR of 3^rd^ instar larval brain and imaginal disc (B–D, light gray) and salivary gland (SG, dark gray ) indicates that the *rpr* and *hid* genes are enriched for the silencing chromatin marks H3K27Me3 (E,F) and H3K9Me3 (G), and have a deficit of the activating mark poly AcH4 (H) in endocycling cells. X-axis: primer position relative to TSS with p53RE in red. Control loci: Act5C, active in both tissue types; Ubx; silenced in SG; heterochromatic locus H23, silenced in B–D and SG. Y-axis is qPCR of pellet DNA normalized to input and either Ubx (E,F), H23 (G), or Act5C (H) levels in B–D. Error bars represent the range of data from two (F–H) or three (E) independent ChIP experiments.

Our previous RT-qPCR data showed that mRNA levels for the pro-apoptotic H99 genes *rpr*, *hid*, *sickle*, and *grim* are much lower in endocycling larval salivary gland (SG) and fat body (FB) tissues than in mitotic cycling larval brain and imaginal discs (B–D) [19]. To test whether this is a result of transcriptional regulation, we examined the promoter activity for two of the genes at the H99 locus, *rpr* and *hid*. The *hid 5′F-WT* (hereafter *hid-GFP)* and *rpr-11-lacZ* reporters contain only the promoter and part of the enhancer regions of the *rpr* and *hid* gene, but lack the transcription units, and therefore are insensitive to post-transcriptional regulation [29], [44]. In untreated animals, the *hid-GFP* reporter was expressed at very low levels in both the mitotic cycling B–D and endocycling SG cells (Figures 1A and 1C). Within four hours after irradiation with 4000 rads of γ-rays, *hid-GFP* was induced to high levels in mitotic cycling B–D cells, consistent with previous reports (Figure 1B) [41], [44]. In contrast, *hid-GFP* expression was not induced by irradiation in the endocycling SG cells (Figure 1D). The *hid-GFP* reporter was also not induced in SG cells 24 hours after radiation, suggesting that both p53-dependent and the delayed p53-independent activation of this reporter is repressed in endocycling cells. Similar results were obtained with the *rpr-11-lacZ* promoter reporter, which was induced by IR in mitotic cycling but not endocycling cells (Figures S1A–S1D). These reporters were also not induced by radiation in other endocycling cells of larval and adult tissues, for example the larval fat body and follicle cells and nurse cells of the adult ovary (Figures S1E–S1H). These results suggest that the block to apoptosis in the endocycling cells acts in part through reduced transcription of the pro-apoptotic H99 genes.

We had previously shown that over-expression of p53 from a *UAS:6xMyc:p53* transgene induces apoptosis in mitotic cycling cells, but it does not in endocycling cells. One possibility is that over-expressed *p53* cannot induce apoptosis in endocycling cells because the checkpoint pathway upstream is uncoupled, and thus p53 may not be activated by Chk2. To address this, we determined whether Chk2 is required for apoptosis in mitotic cycling B–D cells when p53 is over-expressed. Over-expression of *UAS:6xMyc:p53* in B–D cells using an *hsp70:GAL4* driver resulted in comparable levels of apoptosis in Chk2 null and Chk2 wild type animals (Figures S1I–S1L). This result indicates that over-expressed p53 induces apoptosis in diploid cells in the absence of activation by Chk2. This result further suggests that uncoupling of checkpoint signaling upstream of p53 is inadequate to explain the absence of apoptosis in endocycling cells after p53 over-expression.

### Epigenetic silencing at H99 genes in endocycling cells

The results with the *rpr* and *hid* reporters prompted us to ask whether chromatin silencing represses their transcription in endocycling cells. To test this, we performed Chromatin Immuno-Precipitation (ChIP) using antibodies against chromatin silencing and activating marks in mitotic cycling B–D and endocycling SG tissues. Both the *rpr* and *hid* enhancer-promoter regions had higher levels of the silencing mark H3K27Me3 in SG than in B–D cells (SG/B–D ∼10 fold) (Figures 1E and 1F). Within the enhancer-promoter region of both *rpr* and *hid*, H3K27Me3 peaked around the predicted p53 binding sites (p53 response elements, hereafter p53REs) (Figures 1E and 1F). ChIP for another silent chromatin mark, H3K9Me3, showed that it was also higher at *rpr* and *hid* in SG cells (Figure 1G). ChIP for the activating marks poly-acetylated H3 (H3Ac) and H4 (H4Ac) revealed that they were lower at *rpr* and *hid* in SG compared to B–D, although the difference between tissues was not as extreme as for silencing marks (Figure 1H, S2A). Analysis of extant ChIP-array data for H3K27Me3 in salivary glands from the Orr-Weaver lab showed that this silencing mark is heavily enriched across a >400 kb domain spanning the H99 locus (Figure S2B) [45]. Together with the promoter reporter results, these ChIP results suggest that the regulatory regions of the p53 target genes at the H99 locus are epigenetically silenced in endocycling cells.

To further address whether chromatin silences H99 genes, we tested whether RNAi knockdown of epigenetic regulators would sensitize salivary gland cells to p53 over-expression. We created a fly strain that expressed both *UAS:6xMyc:p53* and *UAS:GFP* transgenes under control of the salivary gland driver, *Fkh:GAL4* (Figures S3A-A″, S3C-C″) [46]. We crossed this strain to gene-specific UAS:RNAi strains (200 total, with ∼50% against epigenetic regulators), and screened the living G1 larvae for the appearance of the fluorescent salivary glands. The expression of hairpin RNA corresponding to three different H3K9 histone methyl-transferases (HMT), *Su(var)3-9*, *Eggless* and *G9a*, all resulted in salivary gland cell death [47]–[50]. Knockdown of *Su(var)3-9* was the most severe, with three different RNAi constructs resulting in extremely small salivary glands and high levels of apoptotic cell death as evidenced by pycnotic nuclei, Caspase-3 labeling, and TUNEL (Table 1, Figures S3B-B″). We also observed a more mild, variably-expressive salivary gland cell death after knockdown of *Enhancer of Polycomb (E(Pc))* (Figures S3D-D″). *E(Pc)* is known to modify the silencing of genes by Polycomb complexes, which are writers and readers of the H3K27me3 mark, and is also a suppressor of heterochromatic variegation of genes with the H3K9me3 mark [51]–[53]. Knockdown of *E(Pc)* or the H3K9 HMTs also induced some salivary gland apoptosis in the absence of p53 over-expression, which may be triggered by the constitutive heterochromatic DNA damage that is known to occur in endocycling cells (Figures S3E-E″) [19], [54]. The *E(Pc)* phenotype, however, was clearly more severe when p53 was over-expressed (Figures S3D–E″). These genetic results, combined with the H99 gene expression and ChIP data, are consistent with the idea that chromatin silencing contributes to the repression of apoptosis in endocycling cells.

**Table 1:** Genes recovered in an RNAi screen for salivary gland cell death.

Gene	Gene Function	Knockdown Phenotype^1^
*Su(var)3-9*	H3K9 methyl-transferase	Extremely small gland, faint GFP
*egg*	H3K9 methyl-transferase	Small gland, faint GFP
*G9a*	H3K9 methyl-transferase	Small gland, faint GFP
*E(Pc)*	Novel polycomb member and suppressor of variegation	Small gland, faint GFP
*Nurf-38*	Chromatin Remodeler	Small gland, faint GFP
*Set2*	H3K36 methyl-transferase	Small gland, faint GFP

1 : UAS:RNAi knockdown phenotype of salivary gland in strain with *UAS:6xMyc:p53; Fkh:GAL4,UAS:GFP.*

### Over-expression of p53B, but not p53A, induces apoptosis in endocycling cells

**Figure 2:**
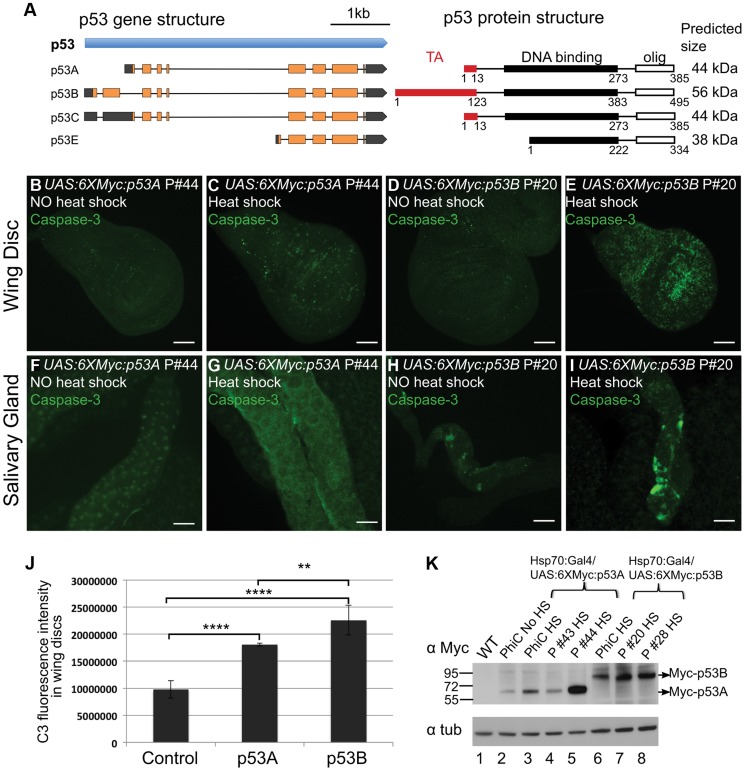
Over-expression of the p53B isoform, but not p53A, induces apoptosis in endocycling cells. (A) Left panel shows a map of the four predicted RNA transcripts for the *Drosophila* p53 gene based on cDNA and RNA-seq data annotated in Flybase. Exons are boxes, with predicted ORFs in orange, and 5′ or 3′ UTRs in gray. The right panel shows predicted protein isoform structures and sizes, with transactivation (TA) and DNA binding domains indicated by red and black boxes respectively. The protein domain predictions are adapted from Khoury and Bourdon (2010), which was based on protein alignment and needed further experimental confirmation [99]. (B–I) Activated Caspase-3 labeling in 3^rd^ instar larval wing discs (B–E) or salivary glands (F–I) after over-expression of *UAS:6XMyc:p53A* (C,G) or *UAS:6XMyc:p53B* (E,I) six hours after a 30 min heat pulse of *hsp70:GAL4* or no heat shock controls (B, D, F, H). Scale bars are 100 microns. (J) Quantification of the fluorescence intensity of Caspase-3 (C3) staining in the larval wing discs. Error bars represent S.E.M. for n = 4. ** p≤0.01, **** p≤0.0001. (K) Anti-Myc Western blot for Myc:p53 protein from adult wild type (WT) and different *UAS:p53* P element (P) or PhiC31 (PhiC) transgenic lines six hours after a 30 min heat pulse of *hsp70:GAL4* (HS), or no heat shock (No HS). Anti-alpha tubulin was used as a loading control.

The *UAS:6XMyc:p53* transgene that we used encodes the 385 amino acid (AA) p53A isoform (hereafter *UAS:6XMyc:p53A*), which has been the most widely studied isoform over the last decade. The current *Drosophila* genome annotation predicts four p53 mRNA transcripts (A,B,C,E), which potentially encode three different protein isoforms (Figure 2A) [55]. The shortest mRNA isoform, p53E, is predicted to encode 334 AA of the p53 C-terminus. The physiological relevance of this protein prediction is in question because RNA-Seq data indicates this mRNA is extremely rare throughout development [55]. The longest 495 AA protein isoform, p53B, differs from p53A by having a 110 AA N-terminal extension that is somewhat conserved with the N-terminal transactivation domain of full-length human p53 (Figure 2A) [56]. RNA-seq data suggests that the p53B mRNA is expressed throughout *Drosophila* development, although at lower levels than p53A mRNA [55].

To more fully understand the role of p53 in the tissue-specific regulation of apoptosis, we transformed fly strains with P-elements carrying the epitope-tagged and GAL4-inducible *UAS:6XMyc:p53A* or *UAS:6XMyc:p53B* isoforms [19]. Within six hours of heat-induced over-expression with *hsp70:GAL4*, both p53A and p53B induced apoptosis in mitotic cycling B–D cells (Figures 2B–E, J). This result is consistent with a recent report which also showed that p53B can induce apoptosis in imaginal discs when over-expressed [57]. We found, however, that p53B over-expression resulted in a significantly larger fraction of apoptotic cells in discs than did p53A (Figures 2C, 2E and 2J). Surprisingly, unlike p53A, the over-expressed p53B isoform was also a potent inducer of cell death in salivary glands (Figures 2F–I). Salivary gland cell death was also observed in *hsp70:GAL4; UAS:6XMyc:p53B* controls without heat induction, which is a result of leaky expression of *hsp70:GAL4* in salivary glands (Figure 2H). Therefore, to compare the relative effect of acute expression of p53A versus p53B in salivary glands, we induced expression of these isoforms using *Sgs3:GAL4*, which first becomes active in mid-3^rd^ instar larval salivary glands. Within hours after this induction, acute p53B expression strongly induced apoptosis in salivary glands while p53A did not (Figure S4 A–B′). Analysis of multiple transformed lines by immunofluorescence and Western blotting showed that while transgene expression was subject to genomic position effect, it did not explain the stronger apoptotic activity of p53B (Figure 2K, Figure S5A–H). In fact, some *UAS:6XMyc:p53A* transgenes were expressed at higher levels than *UAS:6XMyc:p53B*, yet still did not induce apoptosis in endocycling cells (Figure 2K, Figure S5). Transformation of *UAS:6XMyc:p53A* or *UAS:6XMyc:p53B* into the same genomic docking site resulted in similar induced levels of p53A and p53B protein [58], and again showed that over-expressed p53B was a more potent inducer of apoptosis in mitotic cycling and endocycling cells (Figure 2K, Fig. S5I–L).

**Figure 3:**
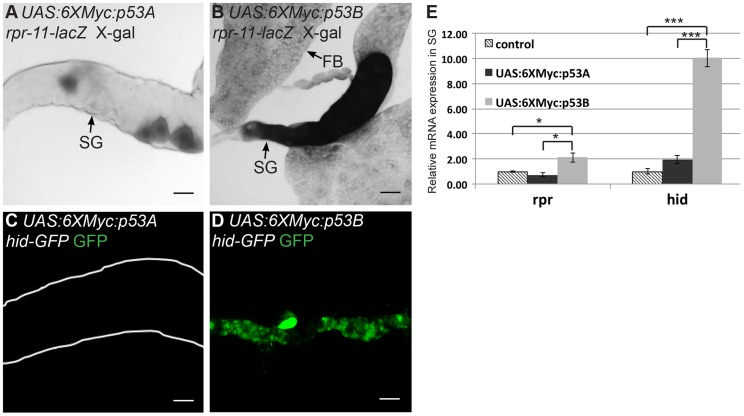
p53B over-expression is a potent inducer of H99 gene transcription. Expression of the *rpr-11-lacZ* (A,B) or *hid-GFP* reporters (C,D) in 3^rd^ instar salivary glands over-expressing either *UAS:6xMyc:p53A* (A,C) or *UAS:6xMyc:p53B* (B,D) six hours after a 30 min heat pulse with *hsp70:GAL4*. SG: salivary gland; FB: fat body. Scale bars are 100 microns. (E) RT-qPCR quantification of endogenous *rpr* and *hid* mRNA in 3^rd^ instar larval salivary gland (SG) cells after over-expression of the indicated transgene or control. Expression was normalized to Act5C, and the level of *rpr* and *hid* in controls was defined as 1. Error bars represent S.E.M. for N = 3. (* p≤0.05, *** p≤0.001).

We next investigated whether p53B over-expression caused salivary gland cell death by inducing H99 gene expression. We first used the *rpr-11-lacZ* and *hid-GFP* reporters. p53B over-expression induced these reporters in both mitotic cycling B-D and SG cells, whereas p53A over-expression induced them only in B–D cells (Figures 3A–3D) [19]. RT-qPCR also indicated that over-expression of p53B, but not p53A, induced transcription of the endogenous *rpr* and *hid* genes in endocycling SG cells (Figure 3E). These data indicate that, when over-expressed, the longer p53B isoform is intrinsically a stronger inducer of H99 gene transcription and apoptosis than p53A in both mitotic cycling and endocycling cells.

### Over-expression of p53B, but not p53A, may activate a paused RNA pol II complex in endocycling cells

**Figure 4:**
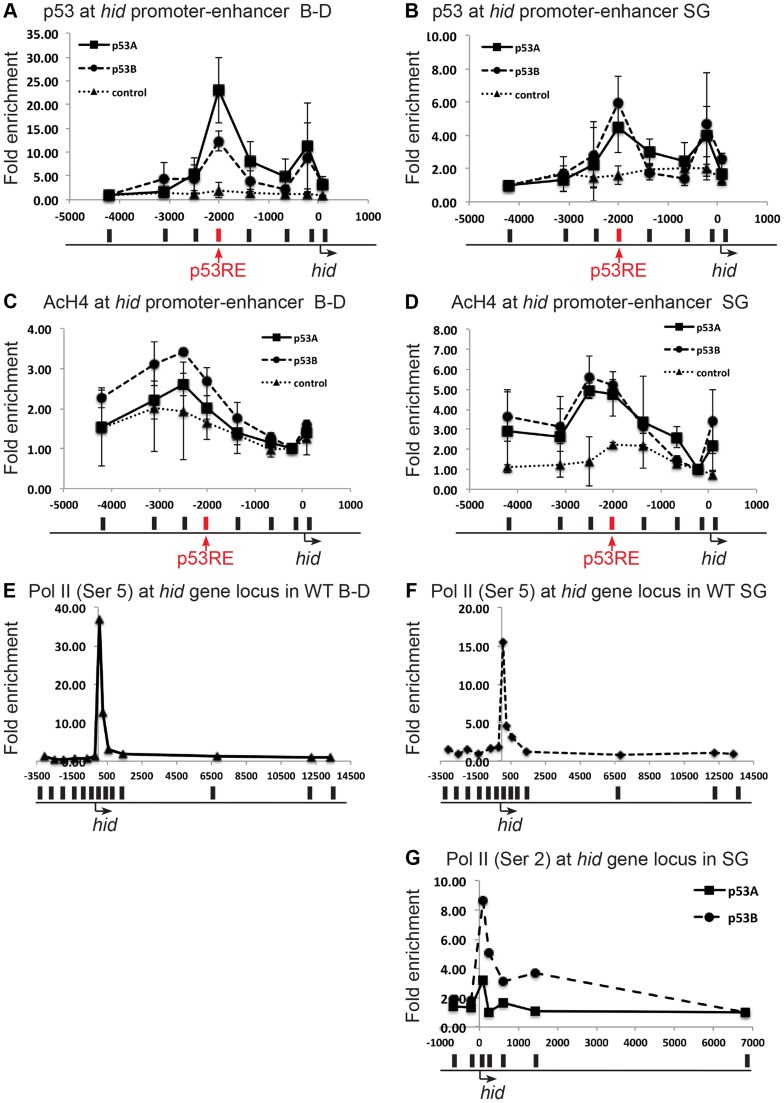
Both over-expressed p53A and p53B bind and recruit acetylation to the *hid* gene, but p53B is better at activating elongation of a paused RNA Pol II. (A, B) Over-expressed p53A or p53B binds to p53REs in *hid* promoter-enhancer in both B–D (A) and SG (B) tissues. ChIP-qPCR analysis with anti-Myc antibody on 3^rd^ instar B–D and SG cells over-expressing *UAS:6xMyc:p53A* (▪), or *UAS:6xMyc:p53B* (•) six hours after a 30 min heat induction with *hsp70:GAL4*, or in controls (▴). X axis: position of the primers relative to the TSS with p53RE in red. Y axis: qPCR value with the −4,000 in *hid* defined as 1. Error bars represent the range of data from two independent biological repeats. (C,D) ChIP-qPCR analysis using anti-poly AcH4 antibody on 3^rd^ instar B–D (C) or SG (D) cells over-expressing either *UAS:6xMyc:p53A* (▪) or *UAS:6xMyc: p53B* (•),six hours after a 30 min heat pulse with *hsp70:GAL4*, or control (▴). X-axis: primer position relative to TSS with p53RE in red. Y axis: qPCR value with the −212 in *hid* defined as 1. Error bars represent the range of two biological replicates. (E, F) A paused RNA Pol II at the hid gene in unchallenged B–D (E) and SG (F) cells. ChIP-qPCR analysis using anti-phosphorylated Pol II Ser5 in 3^rd^ instar B–D and SG cells. X-axis: primer position relative to TSS. Y axis: qPCR values with the +13341 in *hid* defined as 1. (G) p53B is better than p53A for promoting RNA Pol II elongation. ChIP qPCR for elongating RNA Pol II phoshorylated on Serine 2 (Ser 2) at the hid gene in SG cells over-expressing *UAS:6xMyc:p53A* (▪), or *UAS:6xMyc:p53B* (•) six hours after a 30 min heat induction with *hsp70:GAL4.* X-axis: primer position relative to TSS, Y axis: qPCR values with −6810 in *hid* defined as 1. See Figure S6 for similar results at the *rpr* gene.

We further explored the different abilities of p53A and p53B over-expression to induce apoptosis in endocycling cells as an inroad to define the mechanism of apoptotic repression. We first determined if Myc:p53A and Myc:p53B differed in binding to the *rpr* and *hid* genes by using anti-Myc antibody for ChIP experiments. The results indicated that both over-expressed p53A and p53B were bound to the p53REs upstream of the *rpr* and *hid* genes in both the mitotic cycling B–D and endocycling SG cells (Figures 4A and 4B, Figures S6A, B). The level of p53 occupancy at the *hid* promoter, however, was much lower in SG cells than in B–D cells, even though the p53 isoforms were highly over-expressed (Figures 4A, B). This is consistent with the idea that chromatin silencing may act to partly restrict p53 promoter binding in endocycling cells. At the *hid* locus we saw two peaks of binding, one at the previously predicted p53RE at −2,000 and another minor peak around −200 upstream of transcription (Figures 4A, B) [41]. This is the first molecular evidence, to our knowledge, that p53 directly regulates the *hid* gene by binding to this predicted p53RE [38], [41]. Importantly, the relative binding of p53A and p53B was comparable. This data suggested, therefore, that the greater transcriptional potency of p53B relative to p53A in SG cells is not because of differential promoter binding.

We therefore tested whether p53A and p53B differed in their ability to change local chromatin environment in the promoter-enhancer regions of *rpr* and *hid* genes. Chromatin ChIP indicated that over-expression of p53A or p53B in B–D cells increased poly-acetylated H4 levels within the vicinity of the p53RE in the *hid* promoter-enhancer, with p53B inducing a higher level of H4Ac than p53A (Figure 4C). At the *rpr* locus, only p53B increased AcH4 around the p53RE in B–D cells (Figure S6C). These higher levels of acetylation after p53B over-expression correlate with the greater strength of p53B to induce transcription and apoptosis in B–D cells. In the endocycling SG cells, however, p53A and p53B increased histone acetylation to comparable levels at the p53REs in the *rpr* and *hid* genes (Figure 4D, Figure S6D). This suggests that both p53A and p53B may be capable of recruiting HAT co-activators that partially reverse the relatively low level of histone acetylation in SG cells. Therefore, this evidence does not explain the unique ability of p53B to activate transcription in SG cells.

We therefore examined downstream recruitment of RNA pol II to the *rpr* and *hid* promoters. ChIP with an antibody against the initiation form of RNA pol II phosphorylated on serine 5 indicated that in untreated, wild type, control animals there was a high level of RNA Pol II occupancy around the TSS of the *rpr* and *hid* gene in both mitotic cycling B–D and endocycling SG cells, although occupancy was lower in SG cells (Figures 4E, F, Figures S6E, F). These data suggest that there is a paused RNA Pol II complex at the *rpr* and *hid* genes even in the absence of genotoxic stress. Activation of this paused RNA Pol II may mediate an immediate response to genotoxic stress in B–D cells, consistent with the previously observed rapid transcriptional induction of these genes after IR [59]. ChIP with antibodies against the elongating form of RNA pol II phosphorylated on serine 2 indicated that over-expression of p53B was stronger than p53A in stimulating movement of RNA polymerase through the body of the *hid* and *rpr* genes, and also increased occupancy of RNA Pol II near the promoter (Figure 4G, and Figure S6G). All together, the mRNA expression and ChIP data suggest that p53B has the unique ability to induce H99 transcription in endocycling cells by promoting the transition from a paused to elongating RNA Pol II.

### Endocycling cells have very low levels of p53 protein

**Figure 5:**
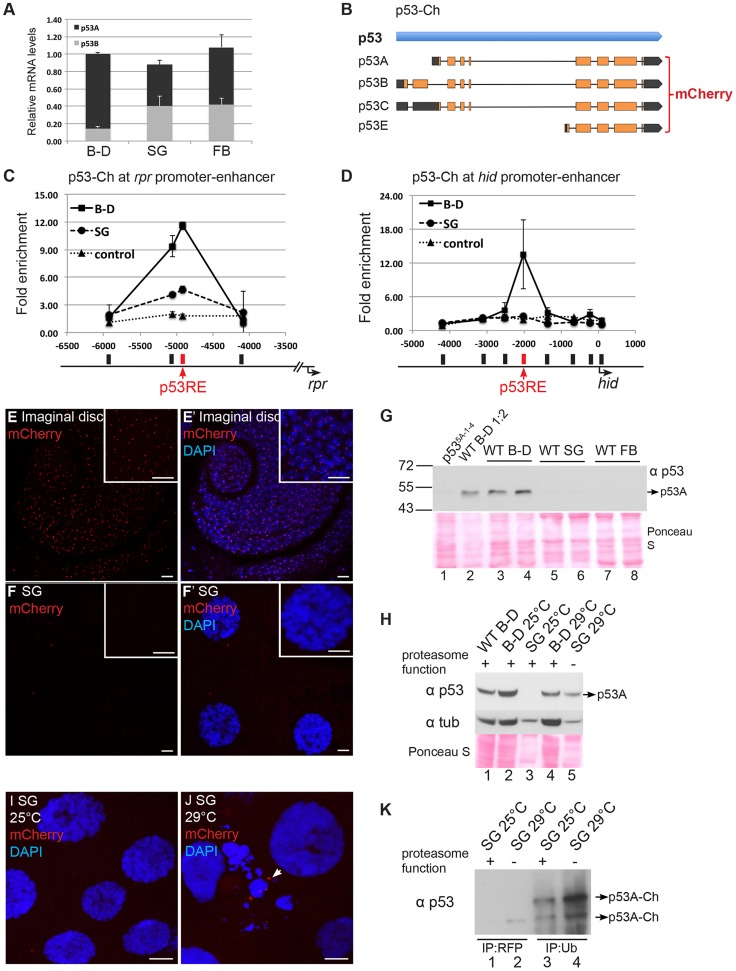
Proteasome-dependent p53 protein degradation in endocycling cells. (A) RT-qPCR quantification of p53 isoform and total mRNA expression levels in different tissue types. Levels were normalized to total mRNA in B–D, which was defined as 1. Error bars represent S.E.M. from three independent experiments. (B) Strategy for tagging p53 BAC with mCherry (p53-Ch) on the common C terminus of all isoforms. (C–D) p53-Ch binds to p53REs in *rpr* (C) and *hid* (D) promoter-enhancer in mitotic but not endocycling tissues. ChIP-qPCR analysis using anti-DsRed antibody on 3^rd^ instar B–D (▪) and SG (•) cells from the *p53-Ch; p53^5A-1-4^* fly strain. The control strain expresses only DsRed (▴). X-axis: primer position relative to TSS with p53RE in red. Y axis: qPCR value with the −6,000 in *rpr* and the −4,000 in *hid* defined as 1. Error bars represent the range of data from two independent biological repeats. (E–F′) p53-Ch fluorescence and DAPI labeling of mitotic cycling cells of 3^rd^ instar larval antenna disc (E,E′) and salivary glands (SG) (F, F′). Scale bars are 10 microns in panels and higher magnification insets. (G) Western blot with anti-p53 to detect endogenous p53 in mitotic cycling larval Brain and Disc (B–D, lanes 1–4), endocycling salivary gland (SG, lanes 5,6) or endocycling fat body (FB, lanes 7,8). The B–D extract in lane 2 was diluted 1∶2. The two lanes for each tissue represent biological replicates with equal amounts of total protein determined by Bradford assay and Ponceau S staining (below). (H) Western blot for endogenous p53 protein from 3^rd^ instar B–D and SG with normal or compromised proteasome function. The temperature-sensitive dominant negative proteasome subunit transgenes (*UAS-pros26^1^; UAS-prosβ2^1^*) were expressed only in the salivary gland using the *Fkh:GAL4* driver. 25°C: permissive temperature for proteasome function. 29°C non-permissive temperature. Equal amounts of total protein were loaded as determined by Bradford assay and Ponceau S staining (below), and blotting for alpha-Tubulin is shown for comparison within the same tissue type. (I,J) p53-Ch foci were not detected in SG cells at permissive temperature for proteasome function (I) but were evident in some SG cells at non-permissive temperature (J arrow). The morphology of some SG nuclei was also aberrant when proteasome function was inhibited (J). (K) p53-Ch protein is ubiquitinated in salivary glands. p53-Ch BAC expression in larvae with the dominant negative proteasome transgenes at permissive (lanes 1,3) or non-permissive (2,4) temperature for proteasome function. Lane 1,2 immunoprecipitation with anti-RFP nanobody, Lane 3,4 immunoprecipitation with anti-ubiquitin antibody, followed by Western blotting with anti-p53. Arrows indicate multiple ubiquitinated p53 species whose abundance increase when proteasome function is compromised.

Our data was consistent with the idea that apoptosis is repressed in endocycling cells through chromatin silencing of H99 genes, which could be overridden by high-level over-expression of p53B. To further explore this idea, we examined the endogenous expression of p53. RT-qPCR indicated that total p53 mRNA levels are similar between mitotic cycling B–D and endocycling SG and FB cells, consistent with our previous RT-qPCR and microarray results (Figure 5A) [19], [39]. Isoform-specific primers showed, however, that p53B mRNA is actually expressed at three fold higher levels in SG and FB than in B–D cells. Therefore, reduced transcription of p53 does not contribute to the repression of apoptosis in endocycling cells.

We next evaluated the levels of p53 protein in different tissues. To potentially detect all the isoforms, we transformed flies with a 24 kb BAC with mCherry fused to the common C-terminus of all the isoforms (p53-Ch). Within this genomic BAC clone, expression of p53 is under control of its normal regulatory sequences (Figure 5B) [60]. Fly strains transformed with the p53-Ch BAC, or an untagged version of the same BAC, rescued the apoptotic response of two different *p53* null mutants (*p53^5A-1-4^* and *p53^11-1B-1^*) in imaginal discs, suggesting that these BACs recapitulate normal p53 function (Figure S7A–D).

We then used a DsRed/mCherry antibody for ChIP to evaluate promoter binding by p53-Ch, which showed that it binds to the p53RE in the enhancer-promoter of both *rpr* and *hid* genes in the mitotic cycling B–D cells, even in the absence of genotoxic stress (Figures 5C and 5D). In contrast, binding of p53-Ch to the *rpr* and *hid* promoters was not detected in endocycling SG cells, even though p53A and p53B can bind to these promoters in SG cells when over-expressed (Figures 5C and 5D).

To gain insight into why p53-Ch promoter binding is not detected in SG cells, we analyzed mCherry fluorescence by microscopy in the *p53-Ch; p53^5A-1-4^* strain. This indicated that p53-Ch was concentrated in 1–2 distinct nuclear foci within B–D cells, which were often in close proximity to the DAPI-bright heterochromatic chromocenter (Figures 5E and 5E′). In larval SG and FB cells, however, p53-Ch fluorescence was not detectible except for very rare, small cytoplasmic puncta (Figures 5F and 5F′). Immunolabeling of fixed cells with the DsRed/mCherry antibody gave similar results. These results suggest that p53 protein levels are low in endocycling cells, explaining why promoter binding is not detected.

**Figure 6:**
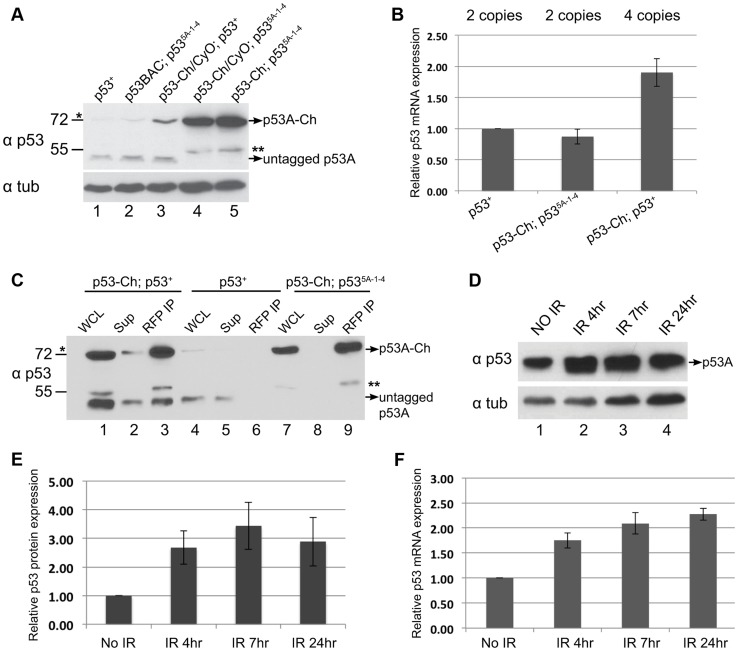
p53 protein complex turnover in mitotic cycling cells. (A) The mCherry tag increases p53-Ch protein in p53 mutant but not wild type mitotic cycling cells. Western blot with anti-p53 to detect endogenous and mCherry-tagged p53 (p53-Ch) protein in 3^rd^ instar B–D extracts. Lane 1: Wild type. Lane 2: Two copies of untagged, wild type p53 BAC transgene in a p53 null mutant background. Lane 3: One copy p53-Ch in a p53 wild type background. Lane 4: One copy p53-Ch in a p53 null background. Lane 5: Two copies p53-Ch in a p53 null background. * indicates a faint non-specific band at ∼72 kDa. ** indicates a degradation product from p53-Ch. Loading control: Anti-alpha Tubulin. (B) RT-qPCR quantification of p53 mRNA expression levels in *p53^+^* or *p53-Ch; p53^5A-1-4^* or *p53-Ch; p53^+^* fly strains. The number of copies for p53 gene is indicated above each genotype. After normalizing to Act5C, the mRNA levels in *p53^+^* were defined as 1. Error bars represent S.E.M from three independent biological replicates. (C) p53-Ch associates with endogenous p53. RFP-nanobody IP from 3^rd^ instar B–D extract of the indicated genotypes followed by Western blot with anti-p53. Lane 1,4,7: whole cell extract (WCL), Lane 2,5,8: supernatant (sup) after IP depletion. Lane 3,6,9: IP pellet. * indicates a faint non-specific band at ∼72 kDa. ** indicates a degradation product from p53-Ch. (D) p53 protein level in mitotic cycling B–D cells increases several fold after IR. Western blot with anti-p53 to detect endogenous p53 in 3^rd^ instar B–D extracts prepared at different time points after IR. Loading control: Anti-alpha Tubulin. (E) Quantification of p53 protein level change in B–D cells at different time points following IR. After normalizing to loading control alpha-tubulin, p53 protein levels from the No IR control was defined as 1. Error bars represent S.E.M from three independent biological replicates. (F) RT-qPCR quantification of p53 mRNA expression level changes following IR. After normalizing to Act5C, p53 mRNA level from No IR sample was defined as 1. Error bars represent S.E.M from three independent biological replicates.

To evaluate the tissue-specific abundance of endogenous p53 protein isoforms, we examined their protein levels by Western Blotting. Antibody raised against the conserved C-terminus of human p53 recognized *Drosophila* p53, and indicated that the most abundant isoform in B–D cells is ∼48 kDa, close to the predicted 44 kDa size of the p53A isoform (Figure 5G, lanes 1, 2, 3, 4) [61]. The predicted 56 kDa p53B and 38 kDa p53E isoforms were not detected in B–D cells. Western blotting of p53-Ch confirmed that there is one major isoform in B–D cells corresponding to the predicted p53A, which is shifted to higher molecular weight by the mCherry tag (Figure 6A). Importantly, none of these isoforms were detected in either SG or FB endocycling cells (Figure 5G, lanes 5, 6, 7, 8). These results showed that p53A is the major isoform in B–D cells, and confirmed that all p53 protein isoforms are expressed at extremely low levels in endocycling SG and FB cells.

The difference between the p53 mRNA and protein levels raised the possibility that p53 protein may be targeted for degradation in endocycling cells. To test this, we inhibited proteasome function in larval salivary glands by using *Fkh:GAL4* to drive expression of two temperature-sensitive, dominant-negative subunits of the proteasome, *P{UAS-Pros26^1^}* and *P{UAS-Prosbeta2^1^}*, created by the Belote lab [62]. We then compared the levels of p53 protein in B–D and SG cells from the same animals at the permissive (25°C) and non-permissive (29°C) temperature for proteasome function in SG cells. Although alpha-Tubulin was used as a loading control, it is only appropriate for comparison within the same tissue type and not between different tissues, and, therefore, Bradford quantification and Ponceau S staining were also used as normalization controls. The results indicated that p53A protein increased to detectable levels in SG cells when proteasome function was compromised at 29°C, but p53B and the other isoforms remained undetectable (Figure 5H). It is important to stress that proteasome function was only inhibited in SG cells, and that expression in B–D cells from the same animals is shown for comparison only (Figure 5H). Immunofluorescence and Western blotting indicated that levels of the tagged p53A-Ch isoform protein also increased in SG cells when proteasome function was compromised (Figure 5I, J, K lane 1,2). This also showed that inhibition of proteasome function had a pleiotropic effect on SG cell morphology (Figure 5J). Immunoprecipitation with an antibody against ubiquitin followed by Western blotting for p53 indicated that the p53A-Ch isoform is ubiquitinated, including higher molecular weight ubiquitinated forms, which increased when proteasome function was compromised at 29°C (Figure 5K, lane 3,4). Taken together, these data indicate that p53A protein is ubiquitinated and degraded by the proteasome in endocycling cells.

### p53 complexes are turned over in mitotic cycling cells

The results clearly indicated that the steady-state level of p53A protein was higher in B–D cells than SG cells, but the p53B and other predicted isoforms were undetectable in both tissue types. Upon further analysis of p53 in B–D cells, however, we noticed that the level of the tagged p53A-Ch isoform was elevated compared to untagged p53A that was encoded either by the endogenous locus or untagged BAC transgenes (Figure 6A, compare lanes 1, 2 with 4, 5). RT-qPCR analysis of B–D cells indicated that mRNA levels for the tagged and untagged p53 was similar (Figure 6B). One interpretation is that p53A protein is also degraded in mitotic cycling B–D cells, although not to the extent it is in endocycling cells, and that the epitope tag at least partially impairs this degradation. In support of this interpretation, we observed that the tagged p53A-Ch isoform was stabilized in B–D cells only in the p53 null mutant background, but not when the endogenous p53 locus was wild type, a difference that is not due to mRNA levels (Figures 6A compare lane 3 with 4, 5, Figure 6B). This suggests that in the p53 mutant background p53-Ch homomeric complexes are stabilized, whereas in the p53 wild type background complexes comprised of p53-Ch and untagged p53 are turned over similar to wild type complexes. Consistent with this, IP with a highly-efficient, single-chain nanobody against RFP/mCherry followed by Western blotting with p53 antibodies indicated that tagged and untagged p53A isoforms are present in the same complex *in vivo* (Figure 6C). These results imply that the wild type untagged p53A exerts a trans-degradation effect on p53A-Ch, and that p53 tetramers are subject to proteolytic turnover in mitotic cycling cells.

In human cells, p53 protein is constantly degraded, but this degradation is inhibited in response to genotoxic stress. We did observe ∼3 fold increase in p53A protein levels in B–D cells at different time points after IR (Figures 6D, E). This increase, however, was proportional to an increase in p53A mRNA after irradiation, consistent with previous reports (Figure 6F) [38], [63]. Thus, there is no evidence that *Drosophila* p53 protein is stabilized in response to genotoxic stress.

## Discussion

**Figure 7:**
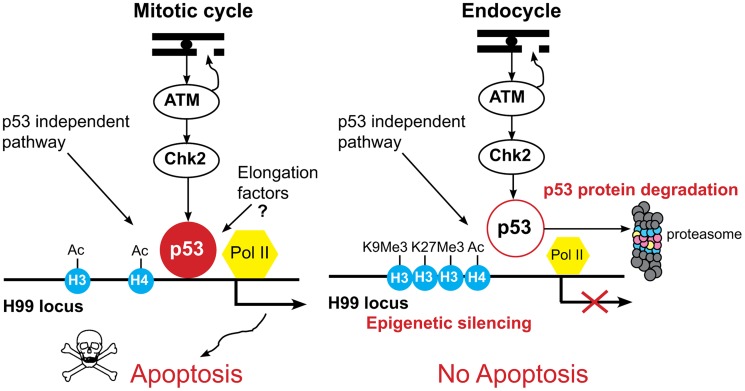
Model for tissue-specific apoptotic responses in *Drosophila*. In mitotic cycling cells, genotoxic stress activates ATM kinase to phosphorylate Chk2, which in turn phosphorylates p53, resulting in transcriptional activation of the H99 genes. Our data suggest that p53 protein stability in these cells is regulated and tunes a threshold level of p53A protein that induces apoptosis only in response to stress. In addition, our data indicate that p53A protein and a paused RNA Pol II are bound to the H99 promoters in the absence of stress. Preloading of p53A and RNA Pol II may prepare these promoters for a rapid stress response. At later time points, p53-independent pathways also mediate cell death through transcriptional activation of H99 genes. In the endocycling cells, ATM kinase is activated, but p53 protein levels are low, p53A is targeted for degradation by the proteasome, and the promoter-enhancer regions of the H99 genes are epigenetically silenced, further blocking their activation by p53-dependent as well as p53-independent cell death pathways. Together, these mechanisms enforce a tight repression of the apoptotic response to genotoxic stress in endocycling cells.

We have used *Drosophila* as a model system to define the molecular mechanisms for tissue-specific apoptotic responses to genotoxic stress. Our data suggest that *Drosophila* endocycling cells repress the apoptotic response in two ways: low level expression of the p53 transcription factor and epigenetic silencing of the p53 target genes at the H99 locus (Figure 7). In mitotic cycling B–D cells, the major p53 protein isoform is p53A, and we did not detect expression of the other predicted p53 protein isoforms. In endocycling SG and FB cells, all of the p53 protein isoforms, including p53A, were below the level of detection. Our data suggest that, similar to human p53, *Drosophila* p53A is ubiquitinated and degraded by the proteasome in endocycling cells. Over-riding this proteolysis by forced expression of p53A did not activate H99 gene transcription or apoptosis in endocycling cells. Together with our other data, these results suggest that downstream chromatin silencing of the H99 locus represses apoptosis in endocycling cells even when p53A protein is abundant. In contrast, we found that over-expression of the longer p53B isoform induced H99 gene expression and apoptosis in endocycling cells. However, the normal physiological expression of p53B protein and binding to the H99 locus was undetectable in endocycling cells, suggesting that the low level of expression of this isoform also contributes to the repression of apoptosis. In the absence of genotoxic stress, we found a paused RNA Pol II at the H99 gene promoters in both mitotic cycling and endocycling cells. In endocycling cells, this paused RNA Pol II complex is activated only when the longer p53B isoform is highly over-expressed. This result implicates polymerase activation as one step that is blocked after DNA damage or p53A over-expression. In mitotic cycling cells, both paused RNA pol II and p53A protein are bound to H99 promoters in the absence of stress, which may prepare cells for a rapid apoptotic response to DNA damage. In addition, our data suggest that p53A protein levels are regulated in mitotic cycling cells, which likely ensures that apoptosis occurs only in response to stress. Together, our results have revealed new mechanisms by which different cells in development modulate their apoptotic response.

### Human and *Drosophila* p53 proteins are targeted for degradation at the proteasome

Previous evidence suggested that *Drosophila* p53 is regulated primarily by Chk2 phosphorylation and not protein stability [26], [59], [64]. Consistent with this, we found that in mitotic cycling cells p53A protein levels do not increase during the early response to radiation, a time when H99 genes are highly induced. At later times after irradiation, p53A protein levels increased only 2–3 fold, a magnitude that is proportional to the increase in p53 mRNA levels, as has been previously reported [38], [43], [63]. Therefore, there is no evidence that the protein stability of p53A or other p53 isoforms changes in response to genotoxic stress. Both with and without genotoxic stress, the cellular levels of p53A protein were relatively low in mitotic cycling cells, and we observed that the epitope tag on p53-Ch increased the abundance of p53A protein in p53 mutant but not p53 wild type cells [59]. A cogent model is that the epitope-tag on p53-Ch partially interferes with p53A proteolysis in mitotic cycling cells, and that untagged p53 can promote the degradation of tagged p53-Ch in the same tetramer. Dampening of p53 protein levels may be critically important to prevent inappropriate apoptosis in the absence of stress. Consistent with this idea, we found that elevated levels of p53A or p53B protein were sufficient to induce apoptosis in mitotic cycling cells even in *Chk2* null animals. We propose that regulation of p53 protein levels in mitotic cycling cells tunes a threshold level of p53 protein that is poised to rapidly activate H99 gene expression when phosphorylated by activated Chk2 in response to DNA damage.

In endocycling cells, however, we were unable to detect any of the p53 protein isoforms using a variety of methods. This tissue-specific regulation of p53 protein abundance is post-transcriptional because mRNA levels were similar between mitotic cycling and endocycling cells. This low level of p53 protein suggests that either its translation is repressed and/or that it is more efficiently proteolyzed in endocycling cells. We favor a model wherein it is p53 proteolysis that is regulated in endocycling cells (Figure 7). In support of this model, compromising proteasome function elevated p53A protein levels in salivary glands. Moreover, p53A is ubiquitinated in endocycling cells, and these modified forms increase when proteasome function is compromised, which is consistent with previous data that p53 turnover is regulated by ubiquitination in *Drosophila* S2 cells [65]. In contrast, the longer p53B isoform remained undetectable when the proteasome function was reduced. Given that proteasome function was only partially compromised, our inability to detect p53B may reflect a more efficient degradation of this longer isoform. This idea is consistent with the known correlation between transactivation domains and ubiquitin-mediated proteolysis for mammalian p53 and other proteins [66].

Although our results suggest that at least the p53A isoform is modified and targeted for degradation by a ubiquitin ligase, the identity of this ligase is unknown. The *Drosophila* genome does not have an obvious ortholog of the ubiquitin ligase MDM2, which targets p53 for degradation in mammalian cells [67]–[70]. It remains possible that another family of ubiquitin ligases mediate p53 degradation in endocycling cells [71], [72]. Nonetheless, our results indicate that regulation of p53 is more similar between flies and humans than previously suspected, a finding that is interesting in the context of growing evidence for conserved p53 functions in flies and humans, including the response to hyperplasia [73].

### p53 target genes at the H99 locus are repressed in endocycling cells

Our data suggest that apoptosis in endocycling cells is repressed in part through chromatin silencing of the pro-apoptotic genes at the H99 locus (Figure 7). Our evidence for silent chromatin marks H3K9me3 and H3K27me3 at H99 are consistent with cytogenetic observations that the H99 chromosome region (75C) is a highly-condensed constriction on salivary gland polytene chromosomes, and genome-wide studies that showed that H3K27me3 is enriched at H99 relative to other loci in salivary glands [45], [74], [75]. Although our genetic data indicate that knockdown of the writers and readers of H3K9me3 and H3K27me3 results in salivary gland apoptosis, it remains possible that knockdown of these regulators causes other types of stress that triggers apoptosis. It is important to note, however, that our results in endocycling cells are also consistent with a previous analysis that indicated that chromatin silencing at H99 dampens the apoptotic response during late embryogenesis [76].

It was previously shown that the chromatin organization at the H99 locus impedes its DNA replication in endocycling cells [45], [74], [77], [78]. As a result, DNA at this locus is not duplicated every endocycle S phase, resulting in a final lower DNA copy number relative to euchromatic loci. This “under-replication” is not the cause of apoptotic repression because we found that in *Suppressor of Underreplication (Su(UR))* mutants, in which the H99 locus is almost fully replicated, endocycling SG cells still did not apoptose in response to DNA damage [45], [77], [79].

Our data suggest that the apoptotic response to genotoxic stress is repressed in endocycling cells because paused RNA Pol II is not activated at *rpr* and *hid* genes (Figure 7). One possibility is that chromatin silencing in endocycling cells restricts recruitment of transcription elongation factors to H99 promoters. We found that over-expressed p53A and p53B were similar in binding and recruitment of acetylation to *rpr* and *hid* promoters, but only p53B activated transcription and apoptosis in endocycling cells. This difference between p53A and p53B isoform activity is attributable to an additional 110 AA amino- terminal transactivation domain in p53B that is somewhat conserved with human p53 [56]. The N-terminus of over-expressed p53B, therefore, may bypass silencing of the H99 genes in endocycling cells by activating this paused RNA polymerase to promote transcriptional elongation. The normal biological function of these paused RNA pol II complexes may be to coordinate a rapid response to developmental signals that trigger apoptosis and autophagy of endocycling larval tissues during metamorphosis [80]–[82].

We propose that low levels of p53 protein and downstream silencing of its target genes both prevent endocycling cell apoptosis (Figure 7). We previously proposed that the apoptotic response to genotoxic stress must be tightly repressed in polyploid endocycling cells because they have constitutive genotoxic stress caused by under-replication of heterochromatic DNA [19], [83], [84]. Consistent with a possible linkage between the endocycle program and apoptotic repression, we recently found that experimentally-induced endocycling cells (iECs) repress apoptosis independent of cell differentiation [25]. It is clear that low levels of p53 protein is not the only mechanism of repression because over-expression of p53A resulted in abundant protein in endocycling cells, but failed to induce H99 transcription or apoptosis. Notably, over-expressed p53 had lower occupancy at H99 promoters in SG than B–D cells, another possible mechanism by which chromatin organization represses apoptosis downstream of p53. Moreover, the complete absence of endocycling cell apoptosis in response to IR suggests that both p53-dependent and p53-independent apoptotic pathways are repressed through silencing of the H99 locus, a point where these pathways intersect. Our data, however, do not rule out the possibility that endocycling cells may use other mechanisms to repress the apoptotic response to DNA damage to ensure their survival despite the continuous genotoxic stress caused by under-replication.

### Mitotic cycling cells are poised to respond to genotoxic stress

In mitotic cycling cells, the p53 protein and paused RNA Pol II were bound to *rpr* and *hid* gene promoters in the absence of stress. This suggests that Chk2 phosphorylation of p53 pre-bound to these promoters activates the paused RNA Pol II to elicit a coordinated and rapid transcriptional response to genotoxic stress [85]. This is consistent with previous evidence that p53-dependent activation of *rpr* and *hid* transcription is readily detectable within 15 minutes of ionizing radiation [59]. This strategy to rapidly respond to stress appears to be conserved to humans where it has been shown that p53 activates paused RNA Pol II at some of its target genes, by indirect or direct physical interaction of p53 with elongation factors [8], [86]. Together, our results suggest that mitotic cycling cells in *Drosophila* are poised to respond to stress by tuning a threshold level of p53 protein that is bound to H99 promoters with a stalled RNA Pol II (Figure 7).

### Cell cycle and tissue-specific regulation of apoptosis

Our data raise the question as to whether similar mechanisms repress apoptosis in mammalian polyploid cells. The transcriptome signatures of fly endocycles is very similar to that of polyploid cycles of mouse liver, megakaryocytes, and placental Trophoblast Giant Cells (TGCs), suggesting a conservation of cell cycle regulation [21], [39], [87]–[91]. It is also known that mouse TGCs do not apoptose in response to UV [92]. Moreover, evidence suggests that p53 protein levels decline when trophoblast stem cells switch into the endocycle and differentiate into TGCs, suggesting that the endocycle repression of apoptosis may be a theme conserved to mammals [93]. The ubiquitin ligase that targets p53 for degradation in TGCs has not been identified, and it is possible that in both *Drosophila* and mouse the same family of ubiquitin ligases targets p53 for degradation in endocycling cells. In addition to developmentally-programmed endocycles, recent evidence suggests that cells can inappropriately switch from mitotic cycles into endocycles, and that this cell cycle switch contributes to genome instability and oncogenesis [23]–[25]. Similar to developmental endocycles, apoptosis may be repressed in these endocycling cancer cells. In support of this idea, recent evidence showed that pro-apoptotic p53 target genes are epigenetically silenced in polyploid cancer cells [94]. Therefore, the mechanisms that repress apoptosis in *Drosophila* endocycling cells may be conserved to humans and relevant to tissue-specific radiation therapy response and oncogenesis [7], [9].

## Materials and Methods

### *Drosophila* genetics

Fly strains were raised at 25°C prior to and during experimental procedures. Fly strains were obtained from the Bloomington Drosophila Stock Center (BDSC, Bloomington, IN, USA) unless otherwise noted. *hid-GFP* fly strain was kindly provided by W. Du. A *y w* strain was used as wild type. For the proteasome dominant negative experiments, crosses were performed at 25°C with 24 hr egg lay. The vials were then transferred to 29°C until 3^rd^ instar larvae.

### Chromatin Immunoprecipitation

The ChIP protocol was modified from previous methods (ChIP-chip protocol for the mod-ENCODE project by Kevin P. White lab and 17–295; Millipore) and entailed at least two biological replicates from separate isolations of 3^rd^ larval instar brains and imaginal discs (B–D) and salivary glands (SG).

### RNA isolation and real-time qPCR

Total RNA was isolated from hand-dissected tissues using TRIzol (15596-026, Invitrogen). 1 µg of RNA from each sample was reverse-transcribed using the QuantiTect Reverse Transcription Kit (Qiagen) according to manufacturer's instructions. qPCR analysis was done on a Stratagene (Santa Clara, CA) Mx3005P machine with SYBR Green Master Mix (600843; Agilent, Santa Clara, CA). For mRNA quantification, Act 5C was used as a reference gene to calculate the relative expression (fold difference). For ChIP-qPCR experiments, the amount of DNA in the pellet was expressed as percentage of input DNA estimated by a standard curve generated from a serial dilution of the input. The values were then normalized to a control. Antibodies used for ChIP are described in Text S1 and PCR primer sequences are listed in Table S1.

### Construction of p53 transgenes


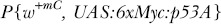
 construction was described previously [19]. 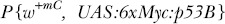 construction was performed using the same strategy. For phiC 31 mediated integration, p53A and B cDNA fragments were cloned into pUAST-w^+^-attB vector and then transformed into the attP docking site at 65B2 (strain 24871) [58]. Unless otherwise stated, the analyses of p53 isoform over-expression were performed using staining corresponding to *UAS:6xMyc:p53A* (P #44) and *UAS:6xMyc:p53B* (P#20).

### BAC recombineering

BAC CH322-178C12 from the P[acman] library was tagged with mCherry at the end of the common last exon of the p53 gene [60], [95]. The resulting construct and untagged BAC were transformed into attP docking sites at 22A3 (strain 24872) using phiC31 integrase.

### Immunoblotting and nanobody immunoprecipitation

Protein extracts were prepared from hand-dissected tissues of mid-late 3rd instar larvae by standard methods using RIPA buffer [96]. For Western blots that compared p53 protein abundance between tissues, we loaded equal amounts of total protein as determined by standard Bradford assay in triplicate using BSA for a standard curve (BSA), and Ponceau S staining, as we have previously reported [39]. For comparison within the same tissue type, mouse anti-α-tubulin was used as loading control. Western blotting was performed as previously described [25], [39]. Antibody dilutions are: mouse anti Myc (9E10, Developmental Studies Hybridoma Bank, University of Iowa) 1∶500, mouse anti p53 (C11, Santa Cruz) 1∶500, mouse anti-α-tubulin (clone DM1A, Sigma) 1∶5,000, and anti-mouse secondary antibody, peroxidase labeled (KPL) at 1∶5,000. The average intensity of the bands for each sample was quantified using ImageJ software.

For RFP nanobody IP [97], 10–15 µl of Chromotek-RFP-Trap beads were added to the protein extracts and incubated for 2 hours at 4°C and precipitated by brief centrifugation. SDS-PAGE sample buffer was added to the washed beads. Mouse monoclonal antibody FK2 (Enzo BML-PW8810) were used to IP for ubiquitin conjugated proteins. 30 µl of Protein A agarose beads were used to pull down the antibody.

### Immunofluorescent microscopy

Mid-late 3^rd^ instar larvae were dissected in either 1× PBS or Grace's solution, and fixed in 6% formaldehyde as previously described [98], and immunolabeled using anti-cleaved-Caspase-3, 1∶50 (Cell Signaling). Secondary antibody was anti-rabbit 488 at 1∶500 dilutions, and DNA was counterstained with DAPI. X-gal staining was performed 6 hours after heat shock treatment as previously described [19]. TUNEL staining (*In Situ* cell death detection kit, TMR red, Roche) was performed according to manufacture's instructions. Wide-field micrographs were taken on a Leica DMRA2 and analyzed using OpenLab (Improvision) software. Confocal micrographs were captured on a Leica SP5 confocal.

### Gamma irradiation

Larvae were irradiated with a total of 4,000 rad (40 Gy) from a Cesium source, and 4, 6 and 24 hours later labeled with anti-activated-Caspase-3.

## Supporting Information

Figure S1 H99 gene promoter activity reporters are repressed in the endocycling cells. (A–D) Expression of the *rpr-11-lacZ* reporter in 3^rd^ instar wing imaginal discs (A,B) and salivary glands (C,D) without (A,C) or with (B,D) IR. X-gal staining was performed 4 hours after 4000 rads of gamma ray treatment. *rpr-11-lacZ* is expressed in no IR controls due to developmental inputs, but expression increases after IR in discs only. (E–H) Expression of the *hid-GFP* promoter-reporter in adult female ovaries without (E,F) or with (G,H) IR. GFP expression is shown in E and G, and corresponding DAPI staining is shown in F and H. *hid-GFP* is induced by IR in mitotic cycling follicle cells before stage 7 (S7), but not in endocycling follicle cells in stage 7 and later egg chambers. (I–J) Over-expression of *UAS:6xMyc:p53A* induces apoptosis in the mitotic cycling cells independent of Chk2 function. p53 over-expression driven by *hsp70:GAL4* in control wing discs from sibling larvae heterozygous (I,J) or homozygous (K,L) for a recessive *Chk2* null mutation. Shown is Caspase-3 staining (I,K), and corresponding DAPI (J,L). Scale bars are 100 microns.
(TIF)

Figure S2 H99 locus has a deficit of activating marks and is enriched for repressive chromatin marks in endocycling cells. (A) ChIP-qPCR of 3^rd^ instar larval brain and imaginal disc (B–D, light gray) and salivary gland (SG, dark gray) indicates that the activating mark poly AcH3 at the promoter-enhancer region of the *rpr* gene is lower in SG than in B–D, whereas acetylation at the Act 5C control locus was similar. X-axis: primer position relative to TSS. (B) Analysis of genome-wide ChIP-array data for H3K27Me3 enrichment in salivary gland cells from Sher et al. paper [45]. The panel shows a signal graph for H3K27Me3 enrichment for an ∼500 kb genomic region centered on the H99 locus (contained within 75C–D region indicated above). The results indicate that H99 resides with an ∼400 kb domain that is enriched for H3K27Me3 compared to the neighboring loci. Genes are annotated below the signal graph. Green bar represents the promoter-enhancer regions of *rpr* and *hid* genes analyzed in Figure 1.
(TIF)

Figure S3 RNAi against epigenetic regulators results in apoptosis in endocycling SG cells. (A-A″) Salivary gland from the screening strain that over-expresses *p53*, *UAS:6xMyc:p53/+ ; Fkh:GAL4, UAS:GFP/+*. (B-B″) Salivary gland from a larva over-expressing *p53* with *Su(var)3-9* knockdown, *UAS:6xMyc:p53/+ ; Fkh:GAL4, UAS:GFP/UAS:Su(var)3-9^RNAi^*. (A, B) GFP fluorescence, (A′, B′) TUNEL, (A″, B″) DAPI. Images in A–B″ were captured at 40× and scale bars are 100 microns. (C-C″) A 10× image of a salivary gland from the screening strain that over-expresses *p53*, *UAS:6xMyc:p53/+ ; Fkh:GAL4, UAS:GFP/+*. (D-D″) Salivary gland from a larva over-expressing *p53* with *E(Pc)* knockdown, *UAS:6xMyc:p53/+ ; Fkh:GAL4, UAS:GFP/UAS:E(Pc)^RNAi^.* (E-E″) E(Pc) knockdown without p53 over-expression, *Fkh:GAL4, UAS:GFP/UAS:E(Pc)^RNAi^.* (C, D, E) GFP fluorescence, (C′, D′, E′) anti-cleaved Caspase 3, (C″, D″, E″) DAPI. Images in C–E″ were all captured at 10× and scale bars are 100 microns.
(TIF)

Figure S4 Acute expression of p53B, but not p53A, isoform induces apoptosis in endocycling cells. (A–B′) Activated Caspase-3 (A, B) and DAPI (A′, B′) labeling in late 3^rd^ instar larval salivary glands after acute expression of *UAS:6XMyc:p53A* (A,A′) or *UAS:6XMyc:p53B* (B,B′) by *SGS3:GAL4* as indicated on the left. Scale bars are 100 microns.
(TIF)

Figure S5 Analysis of multiple strains indicates that the p53B, but not p53A, isoform induces apoptosis in endocycling cells when over-expressed. (A–L) Activated Caspase-3 labeling in 3^rd^ instar larval wing discs (A,B,E,F,I,J) or salivary glands (C,D,G,H,K,L) after over-expression of *UAS:6XMyc:p53A* (A,E,I,C,G,K) or *UAS:6XMyc:p53B* (B,F,J,D,H,L) as indicated on the left. Strains were transformed by either P element transformation into random sites (“P” A–H) or targeted insertion into the same genomic docking site using Phi C31 (“PhiC” I–L). Different numbers #44, #43, #20, #28 indicate independent P element transformants. Tissues were fixed six hours after a 30 min heat pulse of expression using *hsp70:GAL4*. (A–D) are from Figure 2 shown here for comparison. Scale bars are 100 microns.
(TIF)

Figure S6 Both over-expressed p53A and p53B bind and recruit acetylation to the *rpr* gene, but p53B is better at activating elongation of a paused RNA Pol II. (A, B) Over-expressed p53A or p53B binds to p53REs in the *rpr* promoter-enhancer in both B–D (A) and SG (B) tissues. ChIP-qPCR analysis with anti-Myc antibody on 3^rd^ instar B–D and SG cells over-expressing *UAS:6xMyc:p53A* (▪), or *UAS:6xMyc:p53B* (•) six hours after a 30 min heat induction with *hsp70:GAL4*, or in controls (▴). X axis: position of the primers relative to the TSS with p53RE in red. Y axis: qPCR value with the −6,000 in *rpr* defined as 1. Error bars represent the range of data from two independent biological repeats. (C, D) ChIP-qPCR analysis using anti-poly AcH4 antibody on 3^rd^ instar B–D (C) or SG (D) cells over-expressing either *UAS:6xMyc:p53A* (▪) or *UAS:6xMyc: p53B* (•), six hours after a 30 min heat pulse with *hsp70:GAL4*, or control (▴). X-axis: primer position relative to TSS with p53RE in red. Y axis: qPCR value with the −212 in *hid* defined as 1 (see figure 4 C,D). Error bars represent the range of two biological replicates. (E, F) A paused RNA Pol II at the *rpr* gene in unchallenged B–D (E) and SG (F) cells. ChIP-qPCR analysis using anti-phosphorylated Pol II Ser5 in 3^rd^ instar B–D and SG cells. X-axis: primer position relative to TSS. Y axis: qPCR values with −5921 in *rpr* defined as 1. (G) p53B is better than p53A for promoting RNA Pol II elongation. ChIP qPCR for elongating RNA Pol II phoshorylated on Serine 2 (Ser 2) at the hid gene in SG cells over-expressing *UAS:6xMyc:p53A* (▪), or *UAS:6xMyc:p53B* (•) six hours after a 30 min heat induction with *hsp70:GAL4.* X-axis: primer position relative to TSS, Y axis: qPCR values with −6810 in *hid* defined as 1. See Figure 4 for similar results at the *hid* gene.
(TIF)

Figure S7 BAC recombineered p53-Ch rescues p53 null mutant apoptotic response to radiation. (A–D) Anti-Cleaved-caspase-3 staining of 3^rd^ instar larval wing imaginal discs treated with IR. (A) Wild type. (B) *p53^5A-1-4^* null mutant. (C) *p53^5A-1-4^* null mutant with *p53* wild type BAC. (D) *p53^5A-1-4^* null mutant with *p53-Ch* BAC. Scale bars are 100 microns.
(TIF)

Table S1 DNA primers used in this study.
(XLSX)

Text S1 Supplemental materials and methods.
(DOC)
