# Supplementary figures and images for: Low Levels of p53 Protein and Chromatin Silencing of p53 Target Genes Repress Apoptosis in Drosophila Endocycling Cells
Source: PLoS Genet. 2014 Sep 11;10(9):e1004581. doi: 10.1371/journal.pgen.1004581 (PMC4161308; doi:10.1371/journal.pgen.1004581)

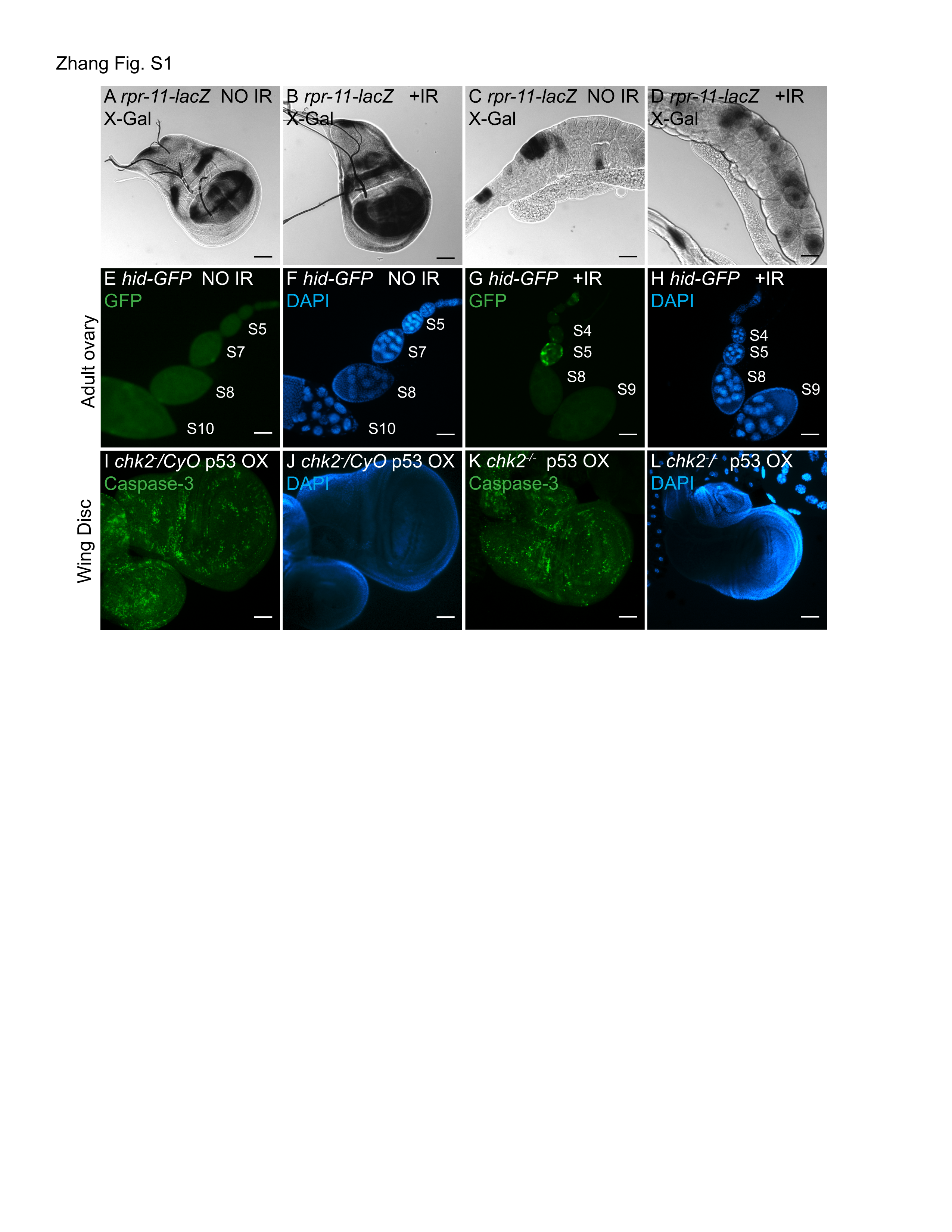

Supplement: Figure S1 — H99 gene promoter activity reporters are repressed in the endocycling cells. (A–D) Expression of the rpr-11-lacZ reporter in 3rd instar wing imaginal discs (A,B) and salivary glands (C,D) without (A,C) or with (B,D) IR. X-gal staining was performed 4 hours after 4000 rads of gamma ray treatment. rpr-11-lacZ is expressed in no IR controls due to developmental inputs, but expression increases after IR in discs only. (E–H) Expression of the hid-GFP promoter-reporter in adult female ovaries without (E,F) or with (G,H) IR. GFP expression is shown in E and G, and corresponding DAPI staining is shown in F and H. hid-GFP is induced by IR in mitotic cycling follicle cells before stage 7 (S7), but not in endocycling follicle cells in stage 7 and later egg chambers. (I–J) Over-expression of UAS:6xMyc:p53A induces apoptosis in the mitotic cycling cells independent of Chk2 function. p53 over-expression driven by hsp70:GAL4 in control wing discs from sibling larvae heterozygous (I,J) or homozygous (K,L) for a recessive Chk2 null mutation. Shown is Caspase-3 staining (I,K), and corresponding DAPI (J,L). Scale bars are 100 microns. (TIF) [file pgen.1004581.s001.tif]

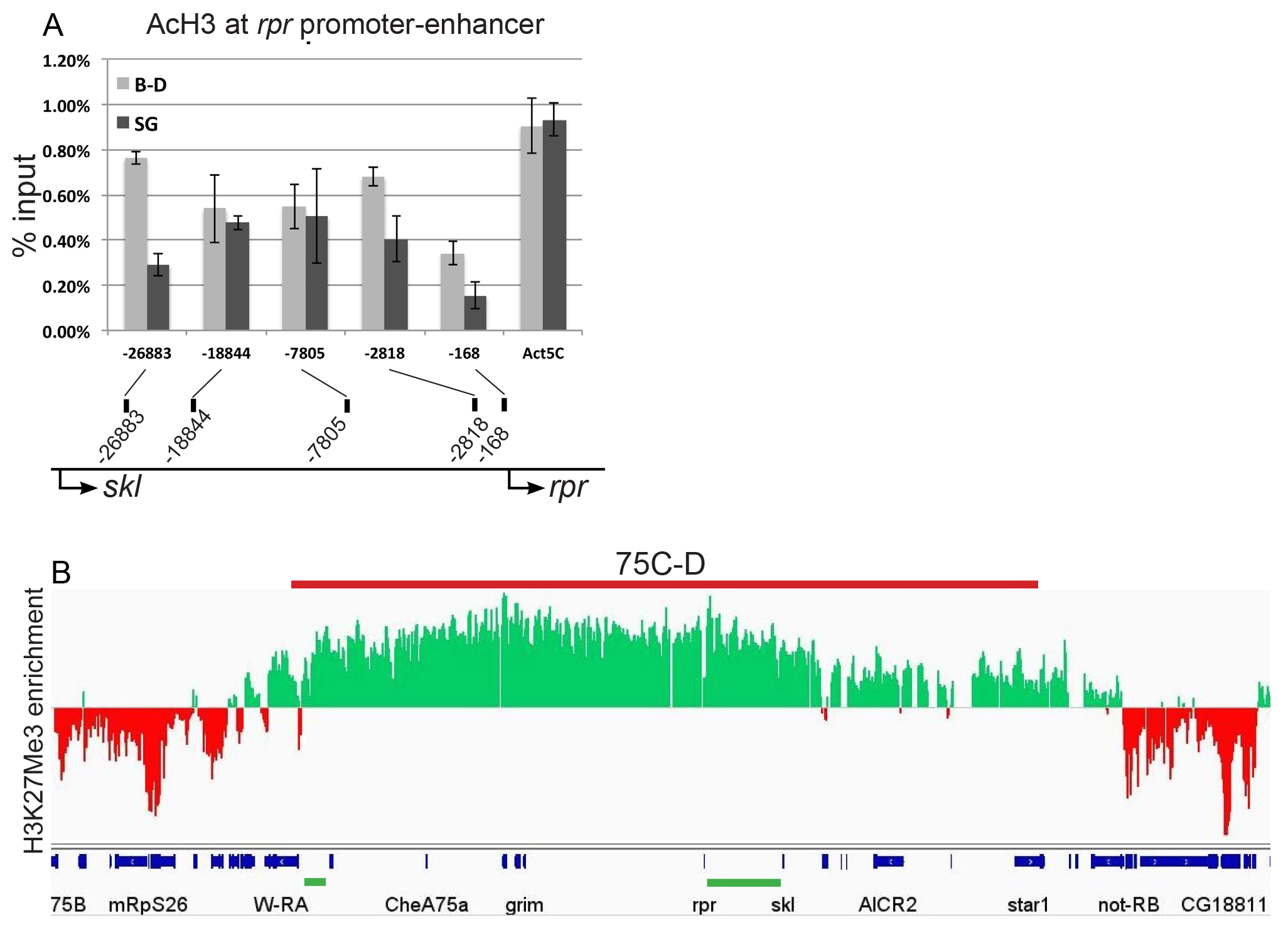

Supplement: Figure S2 — H99 locus has a deficit of activating marks and is enriched for repressive chromatin marks in endocycling cells. (A) ChIP-qPCR of 3rd instar larval brain and imaginal disc (B–D, light gray) and salivary gland (SG, dark gray) indicates that the activating mark poly AcH3 at the promoter-enhancer region of the rpr gene is lower in SG than in B–D, whereas acetylation at the Act 5C control locus was similar. X-axis: primer position relative to TSS. (B) Analysis of genome-wide ChIP-array data for H3K27Me3 enrichment in salivary gland cells from Sher et al. paper [45]. The panel shows a signal graph for H3K27Me3 enrichment for an ∼500 kb genomic region centered on the H99 locus (contained within 75C–D region indicated above). The results indicate that H99 resides with an ∼400 kb domain that is enriched for H3K27Me3 compared to the neighboring loci. Genes are annotated below the signal graph. Green bar represents the promoter-enhancer regions of rpr and hid genes analyzed in Figure 1. (TIF) [file pgen.1004581.s002.tif]

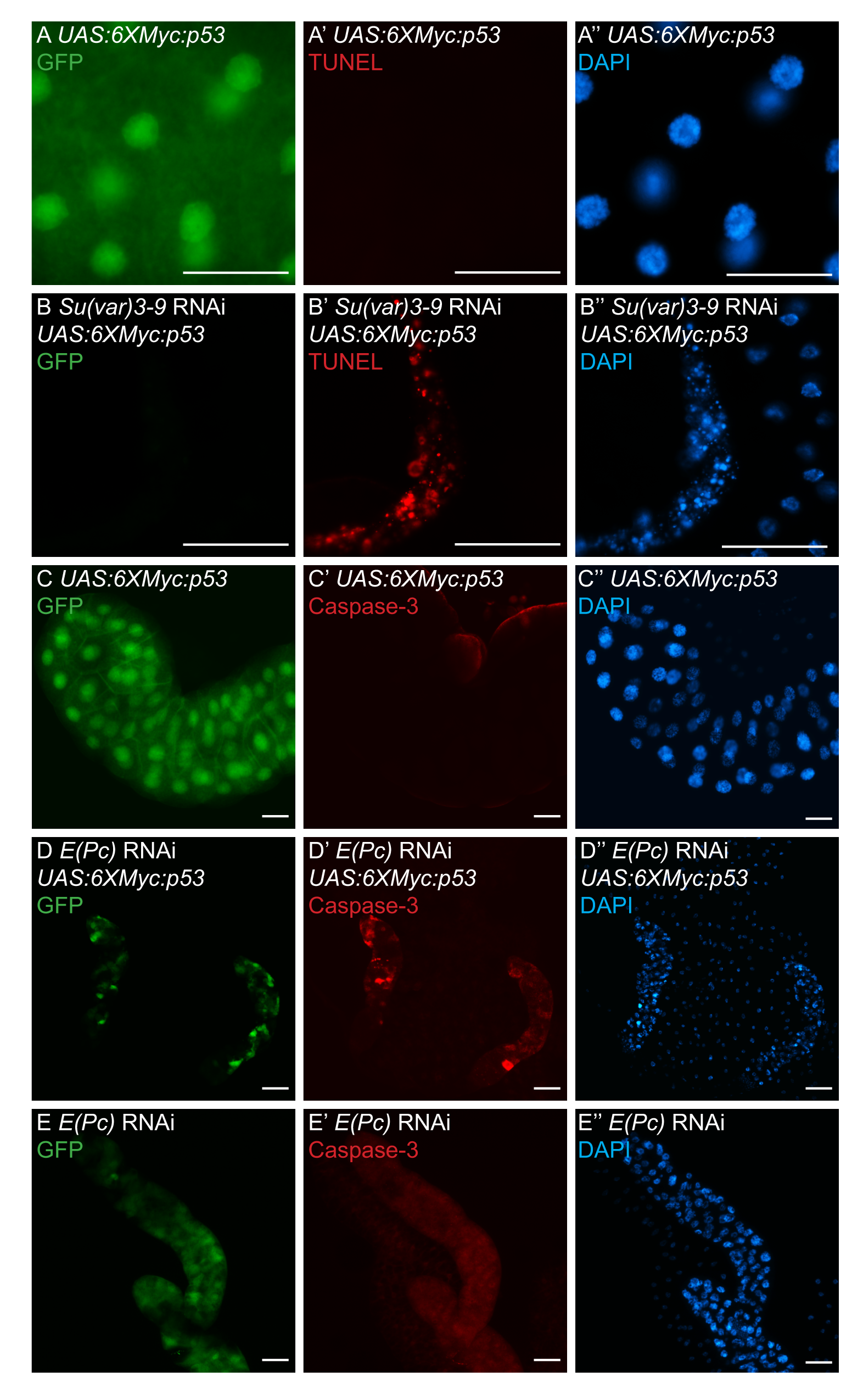

Supplement: Figure S3 — RNAi against epigenetic regulators results in apoptosis in endocycling SG cells. (A-A″) Salivary gland from the screening strain that over-expresses p53, UAS:6xMyc:p53/+ ; Fkh:GAL4, UAS:GFP/+. (B-B″) Salivary gland from a larva over-expressing p53 with Su(var)3-9 knockdown, UAS:6xMyc:p53/+ ; Fkh:GAL4, UAS:GFP/UAS:Su(var)3-9RNAi. (A, B) GFP fluorescence, (A′, B′) TUNEL, (A″, B″) DAPI. Images in A–B″ were captured at 40× and scale bars are 100 microns. (C-C″) A 10× image of a salivary gland from the screening strain that over-expresses p53, UAS:6xMyc:p53/+ ; Fkh:GAL4, UAS:GFP/+. (D-D″) Salivary gland from a larva over-expressing p53 with E(Pc) knockdown, UAS:6xMyc:p53/+ ; Fkh:GAL4, UAS:GFP/UAS:E(Pc)RNAi. (E-E″) E(Pc) knockdown without p53 over-expression, Fkh:GAL4, UAS:GFP/UAS:E(Pc)RNAi. (C, D, E) GFP fluorescence, (C′, D′, E′) anti-cleaved Caspase 3, (C″, D″, E″) DAPI. Images in C–E″ were all captured at 10× and scale bars are 100 microns. (TIF) [file pgen.1004581.s003.tif]

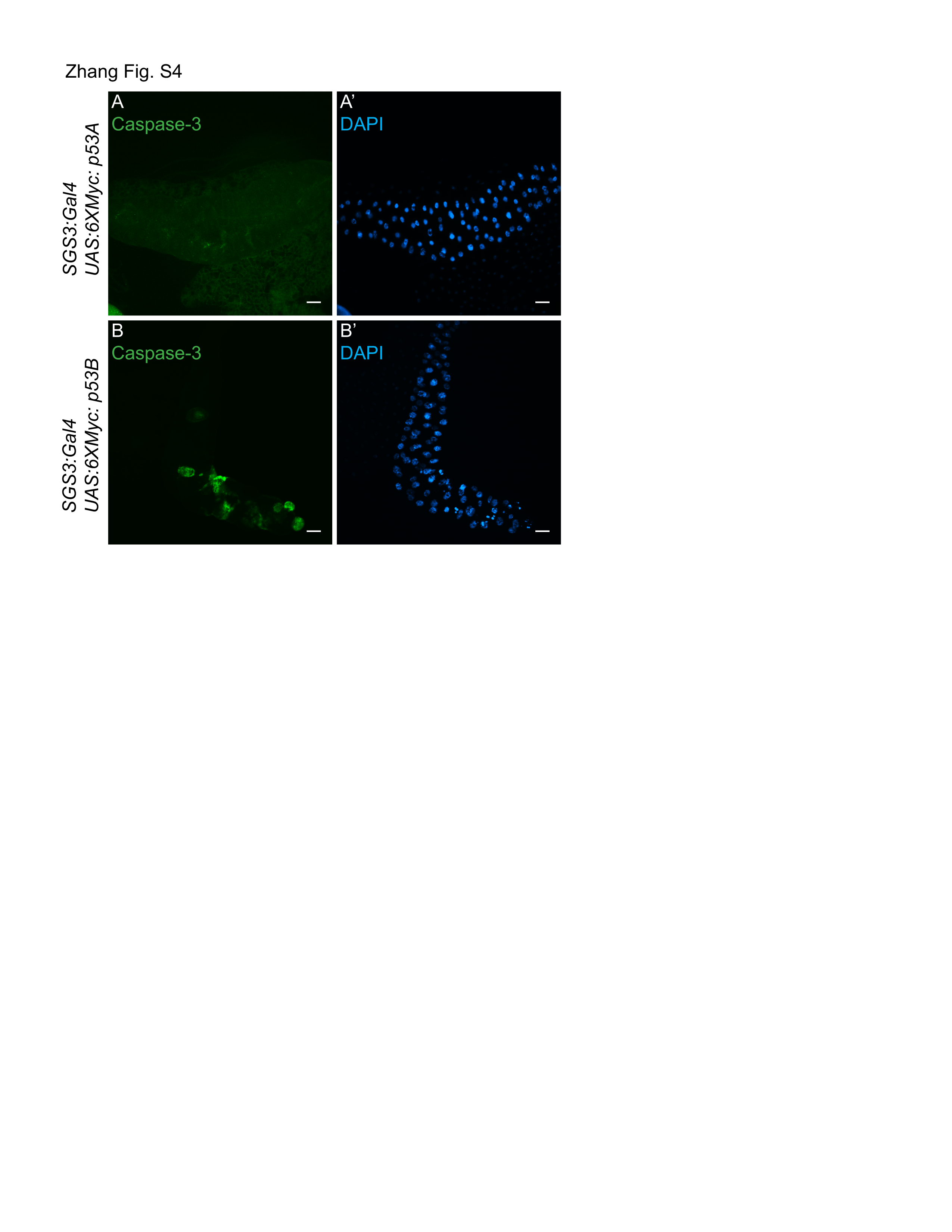

Supplement: Figure S4 — Acute expression of p53B, but not p53A, isoform induces apoptosis in endocycling cells. (A–B′) Activated Caspase-3 (A, B) and DAPI (A′, B′) labeling in late 3rd instar larval salivary glands after acute expression of UAS:6XMyc:p53A (A,A′) or UAS:6XMyc:p53B (B,B′) by SGS3:GAL4 as indicated on the left. Scale bars are 100 microns. (TIF) [file pgen.1004581.s004.tif]

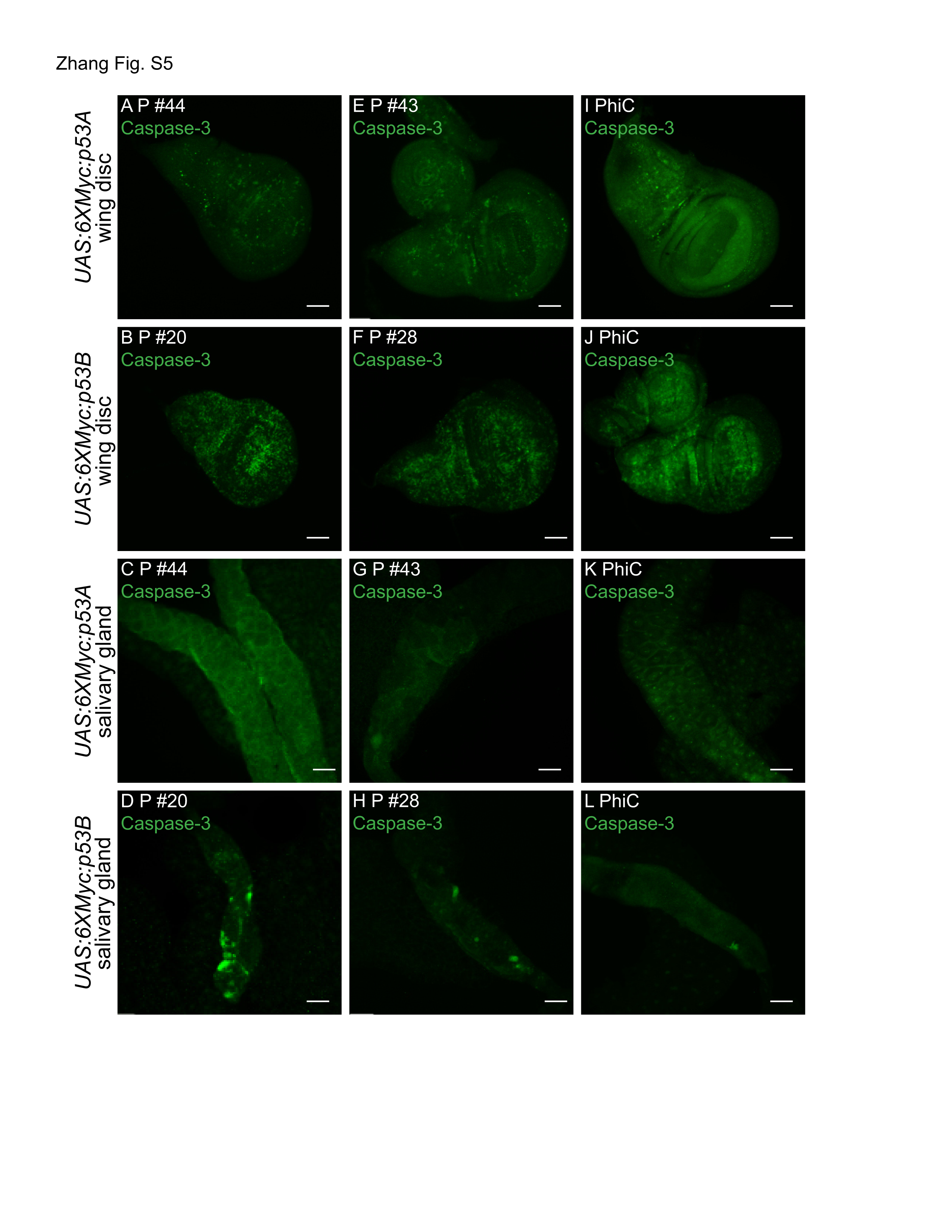

Supplement: Figure S5 — Analysis of multiple strains indicates that the p53B, but not p53A, isoform induces apoptosis in endocycling cells when over-expressed. (A–L) Activated Caspase-3 labeling in 3rd instar larval wing discs (A,B,E,F,I,J) or salivary glands (C,D,G,H,K,L) after over-expression of UAS:6XMyc:p53A (A,E,I,C,G,K) or UAS:6XMyc:p53B (B,F,J,D,H,L) as indicated on the left. Strains were transformed by either P element transformation into random sites (“P” A–H) or targeted insertion into the same genomic docking site using Phi C31 (“PhiC” I–L). Different numbers #44, #43, #20, #28 indicate independent P element transformants. Tissues were fixed six hours after a 30 min heat pulse of expression using hsp70:GAL4. (A–D) are from Figure 2 shown here for comparison. Scale bars are 100 microns. (TIF) [file pgen.1004581.s005.tif]

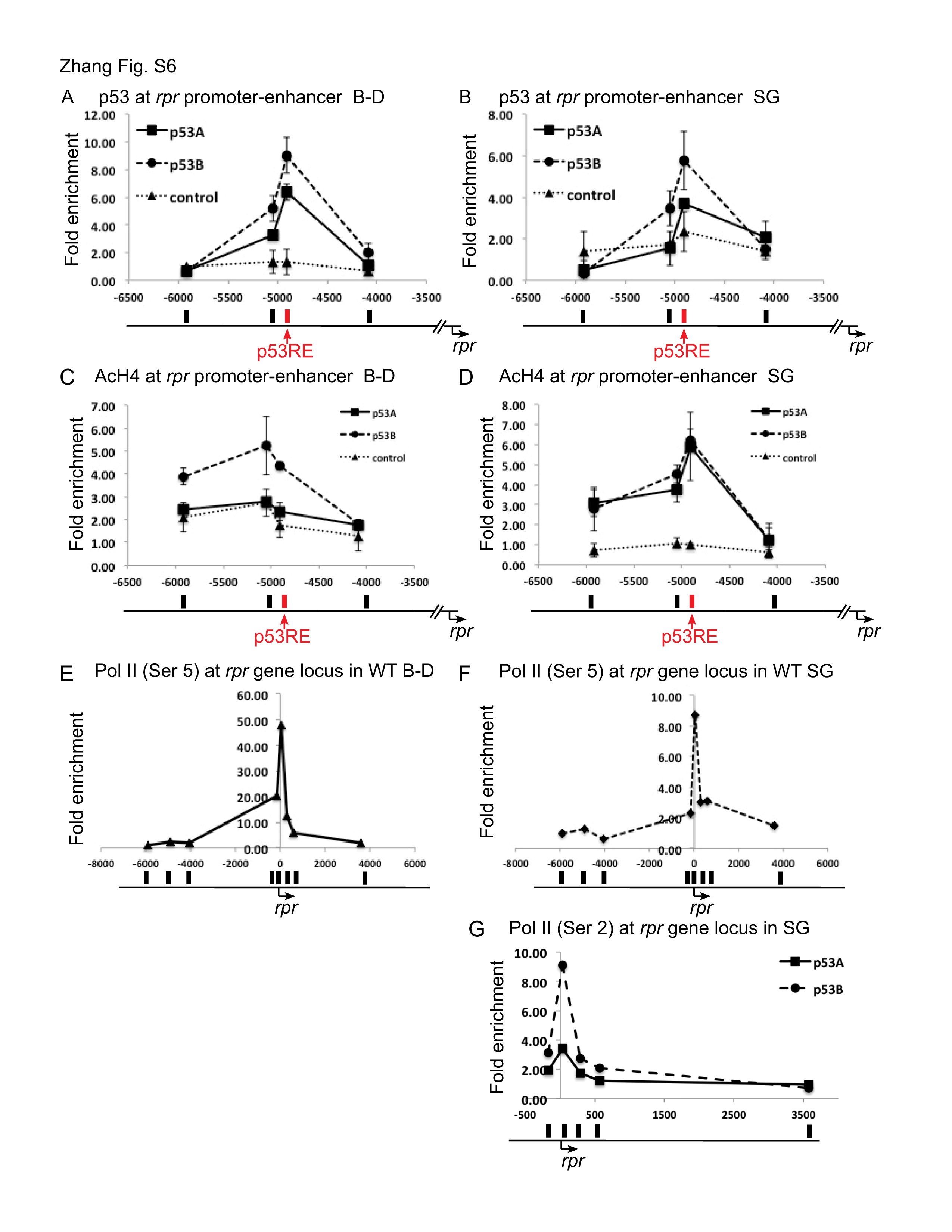

Supplement: Figure S6 — Both over-expressed p53A and p53B bind and recruit acetylation to the rpr gene, but p53B is better at activating elongation of a paused RNA Pol II. (A, B) Over-expressed p53A or p53B binds to p53REs in the rpr promoter-enhancer in both B–D (A) and SG (B) tissues. ChIP-qPCR analysis with anti-Myc antibody on 3rd instar B–D and SG cells over-expressing UAS:6xMyc:p53A (▪), or UAS:6xMyc:p53B (•) six hours after a 30 min heat induction with hsp70:GAL4, or in controls (▴). X axis: position of the primers relative to the TSS with p53RE in red. Y axis: qPCR value with the −6,000 in rpr defined as 1. Error bars represent the range of data from two independent biological repeats. (C, D) ChIP-qPCR analysis using anti-poly AcH4 antibody on 3rd instar B–D (C) or SG (D) cells over-expressing either UAS:6xMyc:p53A (▪) or UAS:6xMyc: p53B (•), six hours after a 30 min heat pulse with hsp70:GAL4, or control (▴). X-axis: primer position relative to TSS with p53RE in red. Y axis: qPCR value with the −212 in hid defined as 1 (see figure 4 C,D). Error bars represent the range of two biological replicates. (E, F) A paused RNA Pol II at the rpr gene in unchallenged B–D (E) and SG (F) cells. ChIP-qPCR analysis using anti-phosphorylated Pol II Ser5 in 3rd instar B–D and SG cells. X-axis: primer position relative to TSS. Y axis: qPCR values with −5921 in rpr defined as 1. (G) p53B is better than p53A for promoting RNA Pol II elongation. ChIP qPCR for elongating RNA Pol II phoshorylated on Serine 2 (Ser 2) at the hid gene in SG cells over-expressing UAS:6xMyc:p53A (▪), or UAS:6xMyc:p53B (•) six hours after a 30 min heat induction with hsp70:GAL4. X-axis: primer position relative to TSS, Y axis: qPCR values with −6810 in hid defined as 1. See Figure 4 for similar results at the hid gene. (TIF) [file pgen.1004581.s006.tif]

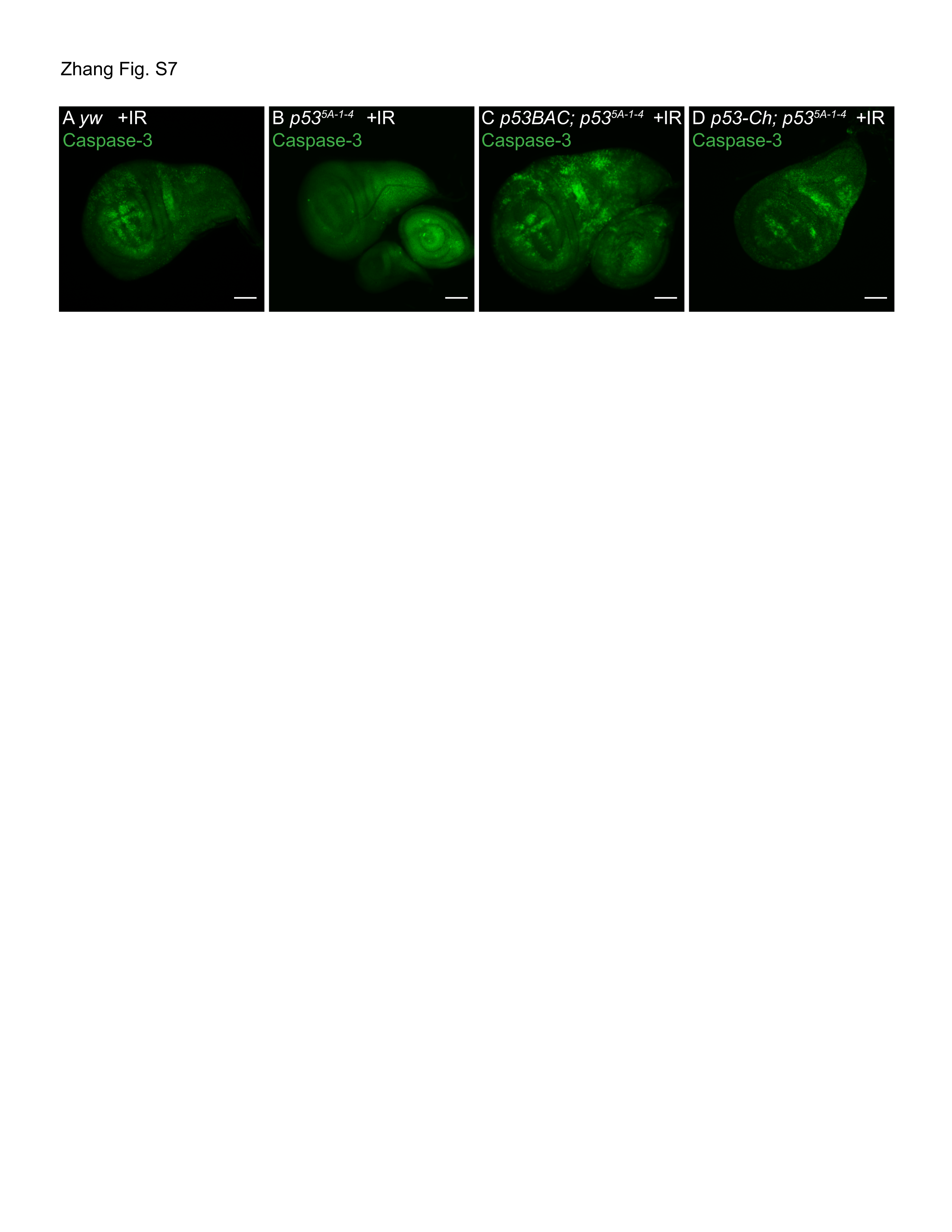

Supplement: Figure S7 — BAC recombineered p53-Ch rescues p53 null mutant apoptotic response to radiation. (A–D) Anti-Cleaved-caspase-3 staining of 3rd instar larval wing imaginal discs treated with IR. (A) Wild type. (B) p535A-1-4 null mutant. (C) p535A-1-4 null mutant with p53 wild type BAC. (D) p535A-1-4 null mutant with p53-Ch BAC. Scale bars are 100 microns. (TIF) [file pgen.1004581.s007.tif]
