# Supplementary material for: Low Levels of p53 Protein and Chromatin Silencing of p53 Target Genes Repress Apoptosis in Drosophila Endocycling Cells
Source: PLoS Genet. 2014 Sep 11;10(9):e1004581. doi: 10.1371/journal.pgen.1004581 (PMC4161308; doi:10.1371/journal.pgen.1004581)
Supplement: Text S1 — Supplemental materials and methods. (DOC) [file pgen.1004581.s009.doc]

**Supplemental Materials and Methods**

**Chromatin Immunoprecitation**

The ChIP protocol was modified from previous methods (ChIP-chip protocol for the mod-ENCODE project by Kevin P. White lab and 17-295; Millipore) and entailed at least two biological replicates from separate isolations of 3^rd^ larval instar brains and imaginal discs (B-D) and salivary glands (SG). In brief, dissected tissues were fixed in bufferA1 (60 mM KCl, 15mM NaCl, 15mM HEPES 7.6, 4mM MgCl_2_, 0.5mM DTT, 0.5% Triton X-100, 1X protease inhibitor cocktail)+1.8% formaldehyde. While fixing, tissues were dounced 3-4 strokes with type A pestle and then washed thoroughly. Cross-linked materials were resuspended in lysis buffer (140mM NaCl, 15mM HEPES 7.6, 1mM EDTA, 0.5mM EGTA, 1% Triton X-100, 0.5mM DTT, 0.1% SDS, 1X protease inhibitor cocktail) and sonicated using a water bath sonicator (Diagenode Bioruptor UCD-200) set on high with 4 rounds of on/off 30s cycles for 5 min each to a modal size of ~450 base pairs. One-tenth of the sample was saved as input, and the remainder was aliquoted for different IPs. Immunoprecipitation was then performed in parallel by adding 2-5 μl (∼2-5 μg) of antibodies, followed by nutation at 4°C overnight. The next day 50 μl of 50% protein A agarose beads (15918-014; Invitrogen) pretreated with sheared salmon sperm DNA was added and incubated 3 h at 4°C and precipitated by brief centrifugation. The beads were washed two times each with low-salt, high-salt, LiCl, and Tris–EDTA buffers. Elution and reversal of cross-linking was done in elution buffer (10 mM EDTA, 1% SDS, 50 mM Tris-Cl pH 8) with heat at 65°C overnight. Both the input and eluate were treated with 1 μg of RNaseA (11119915001; Roche) and 50 μg of proteinase K (P8102S; New England BioLabs). DNA was then purified with standard phenol/chloroform procedure and used for qPCR analysis. Antibodies used for ChIP experiments are: rabbit anti-H3K27Me3 (Millipore 07-449), rabbit anti-H3K9Me3 (Millipore 07-442), rabbit anti-AcH3(Millipore 06-599), rabbit anti-AcH4( Millipore 06-598), mouse anti-Myc (9E10, Developmental Studies Hybridoma Bank, University of Iowa), rabbit anti-DsRed (Clontech 632496), rabbit anti-RNA polII Ser2 (abcam 5095), rabbit anti-RNA polII Ser5 (abcam 5131).

**RNA isolation and real-time qPCR**

Total RNA was isolated from hand-dissected tissues using TRIzol (15596-026, Invitrogen). 1 μg of RNA from each sample was reverse-transcribed using the QuantiTect Reverse Transcription Kit (Qiagen) according to manufacturer’s instructions. cDNA was diluted 5 fold and 1 μl was used for qPCR. qPCR analysis was done on a Stratagene (Santa Clara, CA) Mx3005P machine with SYBR Green Master Mix (600843; Agilent, Santa Clara, CA). Forty cycles of PCR were run with a three-step protocol (denaturation at 95°C for 15s, annealing at 62°C for 20 s and extension at 72°C for 20s). For mRNA quantification, Act 5C was used as a reference gene to calculate the relative expression (fold difference). For ChIP-qPCR experiments, the amount of DNA in the pellet was expressed as percentage of input DNA estimated by a standard curve generated from a serial dilution of the input. The values were then normalized to a control. PCR primer sequences are listed in Table S1.

**Construction of p53 transgenes**

*P(w^+mC^, UAS:6X Myc: p53A)* and *P(w^+mC^, UAS:6X Myc: p53B)* was constructed by PCR amplification of p53A and p53B cDNA from GH11591 and AT28346 cDNA clone obtained from DGRC using primers that encompass the region from the start codon

to the termination codon (Table S1). *P(w^+mC^, UAS:6X Myc: p53A)* construction was described previously [19]. For *P(w^+mC^, UAS:6X Myc: p53B)*, the PCR product was subcloned into pCS2 vector in which 6X Myc epitope tag had been cloned between the EcoR1 and Xho1 sites. This resulted in an in-frame N-terminal fusion of the AUG to 6X Myc epitope tag. The myc-tagged p53B transgene was cloned in p(UAST) vector between the NotI and XhoI sites. For phiC 31 mediated integration, p53A and B cDNA fragments were cloned into pUAST-w^+^-attB vector [58]. Vector DNA was tranformed into attP docking sites at 65B2 (strain 24871) using phiC31 integrase. Microinjection for all constructs was performed by Rainbow Transgenics**.**

**BAC recombineering**

For p53 recombineering, BAC CH322-178C12 from the P[acman] library was chosen. BAC preparation was performed as previously described [60,93] and electroporated into SW102 cells. For recombineering process, three PCR fragments were amplified: upstream fragment containing ~500bp left homology arm to the BAC; insertion fragment containing the fluorescent tag and the selection marker (GalK); and downstream fragment containing ~500bp right homology arm to the BAC. The PCR products were quantified and fusion PCR was used to “stitch” three fragments into a single linear fragment. The resulting linear fragment was electroporated into recombineering competent SW102 cells carrying the original BAC. After electroporation, the cells were recovered, washed and plated on M63 minimal media with galactose to select for GalK positive cells at 32°C. Well-isolated colonies from the plates were confirmed by inoculating onto MacConkey plate. GalK positive cells should turn bright fuchsia due to products from galactose metabolism. In the second recombineering step, 100 bp deletionmer containing 50bp homology each to the right and left of the deletion site was electroporated into the recombineering competent SW102 cells carrying BAC::fluorescent tag+GalK construct. The cells were recovered, washed and plated on minimal media with DOG to select against GalK. The colonies were verified by colony PCR based on the size reduction of GalK fragment and further confirmed by DNA sequencing. The verified plasmid containing BAC::fluorescent tag was mini-prepped and transformed into EPI300 cells for large amount of plasmid preparation. The resulting constructs were transformed into attP docking sites at 22A3 (strain 24872) using phiC31 integrase.

**Immunoblotting**

Mid-late 3rd instar larvae were hand dissected and protein extracts were made from different tissues by standard methods using RIPA buffer [94].

To avoid assumptions about equal gene expression of housekeeping genes in different tissues, we did not normalize to another protein for loading control, but instead normalized to total protein concentration as determined by standard Bradford assay in triplicate using BSA for a standard curve and Ponceau S staining, as we have previously reported [39]. Equal amounts of total protein from different tissues were loaded onto the gel. The average intensity of the bands for each sample was quantified using ImageJ software.

Western blotting was performed using BIO-RAD SDS-PAGE equipments and reagents (BIO-RAD), transferred to Immobilon-P PVDF membrane (Millipore), and stained by Ponceau S for loading control. The membrane was then blocked with 5% nonfat milk in 1x TBST (1x TBS, 0.1% Tween-20). Blots were labeled with primary mouse anti-Myc (9E10, Developmental Studies Hybridoma Bank, University of Iowa) 1:500, mouse anti p53 (C11, Santa Cruz) 1:500 and mouse anti-α-tubulin (clone DM1A, Sigma) 1:5,000, and secondary antibody anti-mouse, peroxidase labeled (KPL) at 1:5,000. Antibodies were diluted in 2% non-fat milk in 1x TBST. The SuperSignal Pico ECL kit (Thermo Scientific) was used to detect peroxidase activity. Blots were exposed on X-ray film and developed using an X-Omat machine (Kodak).
